# Function and Distribution of 5-HT_2_ Receptors in the Honeybee (*Apis mellifera*)

**DOI:** 10.1371/journal.pone.0082407

**Published:** 2013-12-06

**Authors:** Markus Thamm, Daniel Rolke, Nadine Jordan, Sabine Balfanz, Christian Schiffer, Arnd Baumann, Wolfgang Blenau

**Affiliations:** 1 Department of Biochemistry and Biology, University of Potsdam, Potsdam, Germany; 2 Institute of Complex Systems (ICS-4), Research Center Jülich, Jülich, Germany; 3 Institut für Bienenkunde (Polytechnische Gesellschaft), Goethe University Frankfurt, Oberursel, Germany; University of Leipzig, Germany

## Abstract

**Background:**

Serotonin plays a pivotal role in regulating and modulating physiological and behavioral processes in both vertebrates and invertebrates. In the honeybee (*Apis mellifera*), serotonin has been implicated in division of labor, visual processing, and learning processes. Here, we present the cloning, heterologous expression, and detailed functional and pharmacological characterization of two honeybee 5-HT_2_ receptors.

**Methods:**

Honeybee 5-HT_2_ receptor cDNAs were amplified from brain cDNA. Recombinant cell lines were established constitutively expressing receptor variants. Pharmacological properties of the receptors were investigated by Ca^2+^ imaging experiments. Quantitative PCR was applied to explore the expression patterns of receptor mRNAs.

**Results:**

The honeybee 5-HT_2_ receptor class consists of two subtypes, Am5-HT_2α_ and Am5-HT_2β_. Each receptor gene also gives rise to alternatively spliced mRNAs that possibly code for truncated receptors. Only activation of the full-length receptors with serotonin caused an increase in the intracellular Ca^2+^ concentration. The effect was mimicked by the agonists 5-methoxytryptamine and 8-OH-DPAT at low micromolar concentrations. Receptor activities were blocked by established 5-HT receptor antagonists such as clozapine, methiothepin, or mianserin. High transcript numbers were detected in exocrine glands suggesting that 5-HT_2_ receptors participate in secretory processes in the honeybee.

**Conclusions:**

This study marks the first molecular and pharmacological characterization of two 5-HT_2_ receptor subtypes in the same insect species. The results presented should facilitate further attempts to unravel central and peripheral effects of serotonin mediated by these receptors.

## Introduction

The biogenic amine serotonin (5-hydroxytryptamine, 5-HT) has important physiological functions including the regulation of energy balance and food intake, gastrointestinal and endocrine function, and cardiovascular and pulmonary physiology (for a recent review, see: [1]). In the central nervous system (CNS), serotonin contributes to the regulation of appetite, mood, sleep, and cognitive functions, including learning and memory. In humans, impaired serotonin signaling has been implicated in diseases such as migraine, schizophrenia, and depression (for reviews, see: [2], [3]). At present, 14 distinct genes encoding 5-HT receptors have been identified in total in mammals. These proteins have been assigned to seven receptor classes (5-HT_1_ to 5-HT_7_; [4], [5]). Except for 5-HT_3_, which is a ligand-gated ion channel, all mammalian 5-HT receptors belong to the family of G protein-coupled receptors (GPCRs). The 5-HT_1_ and 5-HT_5_ receptors couple preferentially to G_i/o_ proteins and inhibit cAMP synthesis. The 5-HT_2_ receptors activate G_q/11_ proteins, which mediate the hydrolysis of inositol phosphates and cause a subsequent increase in cytosolic Ca^2+^ ([Ca^2+^]_i_). The 5-HT_4_, 5-HT_6_, and 5-HT_7_ receptors couple to G_s_ proteins and promote cAMP formation. Orthologous G protein-coupled 5-HT receptors with conserved signaling pathways have been discovered in protostomes including nematodes [6], crustaceans [7], [8], and insects [9]-[11]. To date, four 5-HT receptor subtypes have been characterized in the fruit fly (*Drosophila melanogaster*). Phylogenetically, they cluster within the mammalian 5-HT_1A_ [12], 5-HT_2_ [13], and 5-HT_7_ [14] subfamilies.

In addition to the genetic model organism *D. melanogaster*, the honeybee (*Apis mellifera*) has greatly contributed to improving the general understanding of serotonergic control and modulation of various types of behavior [11], [15]–[19]. The distribution of serotonergic neurons has been mapped precisely in the CNS of this social insect [20], [21], and the serotonin content in the brain has been determined at various developmental stages [22], [23]. Serotonin levels are not constant during a bee’s lifespan, with older foraging bees having higher serotonin contents than younger bees working in the hive [23]. In single-cohort colonies, which consist of same-aged bees, significant differences in serotonin levels have been found in the antennal lobes of forager compared with nurse bees [24]. Together, these results suggest that differences in serotonin levels are related to specific task(s) that bees perform, rather than to their age.

Several aminergic receptors have been characterized from honeybees in recent years [25]–[31]. However, our present knowledge about the molecular and pharmacological properties of 5-HT receptors remains limited. So far, only a 5-HT_1A_ [32] and a 5-HT_7_ [33] receptor have been uncovered. The aim of this study was the molecular identification and detailed pharmacological characterization of honeybee 5-HT_2_ receptors.

So far, not much is known about the functions of 5-HT_2_ receptors in insects. In *D. melanogaster*, the Dm5-HT_2α_ receptor (CG1056) regulates cellular movements during germband extension and cuticular formation during early embryogenesis [34], [35]. Furthermore, 5-HT_2_ receptors have been implicated in the modulation of the daily activity pattern, anticipatory behavior, and aggression (for a review, see: [19]). Recently, the Cv5-HT_2α_ receptor of the blowfly, *Calliphora vicina*, has been shown to be expressed not only in the brain but also in the salivary gland [36]. Here, its activation by serotonin leads, *via* an increase in [Ca^2+^]_i_, to an elevation of the Cl^−^ permeability of both the basolateral and apical membrane and thus facilitates Cl^−^ movement from the haemolymph into the lumen of the gland [36].

Inspection of the completely sequenced honeybee genome [37] has revealed the existence of two candidate genes encoding 5-HT_2_ receptors: Am5-HT_2α_ and Am5-HT_2β_ [10], [11]. After heterologous expression, both receptors cause increases in ([Ca^2+^]_i_) upon stimulation with nanomolar concentrations of serotonin. These responses are efficiently blocked by 5-HT receptor antagonists, but with subtype-specific patterns of efficacy and potency. Because of their preferential expression in glandular tissues, both 5-HT_2_ receptor subtypes are likely candidates for the control or modulation of important secretory processes in the honeybee.

## Materials and Methods

### Cloning of Am5-ht2 cDNAs

Single *Apis mellifera* drone brains were used to prepare poly(A)^+^ RNA with the Micro-FastTrack™ 2.0 Kit (Invitrogen, Karlsruhe, Germany). Drones possess a haploid genome and, therefore, single nucleotide substitutions in cDNA clones cannot be due to allelic polymorphisms. Synthesis of cDNA employed the AccuScript™ High Fidelity 1^st^ Strand cDNA Synthesis Kit (Stratagene, Amsterdam, Netherlands). Specific primers (Table S1) allowed the entire coding region of the receptors to be amplified. The polymerase chain reaction (PCR) was carried out for 2.5 min at 94°C (1 cycle), followed by 35 cycles of 40 s at 94°C, 40 s at 54°C (*Am5-ht2α*) or 64°C (*Am5-ht2β*), 150 s at 72°C, and a final extension of 10 min at 72°C. PCR products were cloned into pGEM-T vector (Promega, Mannheim, Germany) and subsequently sequenced (AGOWA, Berlin, Germany). The nucleotide sequences of *Am5-ht2α* and *Am5-ht2β* have been submitted to the European Bioinformatics Institute (EBI) database (accession nos. FR727107 and FR727108, respectively).

### Multiple sequence alignment and phylogenetic analysis

Amino-acid sequences used for phylogenetic analysis were identified by protein-protein Basic Local Alignment Search Tool (BLAST) searches of the National Center for Biotechnology Information (NCBI) database with the deduced amino acid sequence of *Am5-ht2α* (Am5-HT_2α_) as “bait”. Values for identity (ID) and similarity (S) were calculated by using the BLOSUM62 substitution matrix in BioEdit 7.0.5. MEGA 4 [38] was used to calculate the genetic distances between the core sequences and to construct maximum parsimony trees with 2000-fold bootstrap re-sampling. The *D. melanogaster* rhodopsin 1 (*ninaE*) and FMRFamide receptor sequences were used as out-groups.

### Quantitative real-time PCR

Samples from various tissues of individual honeybee workers and drones were collected, immediately frozen in liquid nitrogen, and stored at -80°C until use. Total RNA was extracted by using the RNeasy Mini Kit (Qiagen, Hilden, Germany) and served as the template for cDNA synthesis. From each sample, two independent cDNA syntheses were performed by using SuperScriptIII (Invitrogen) according to the manufacturer’s instructions. Quantitative real-time PCR (qPCR) was carried out on a Rotor Gene Q (Qiagen) by using TaqMan technology with various fluorescent dyes to allow duplex measurements of receptor and reference gene expression. The fluorescent dyes used as 5’modifications were 6-FAM-phosphoramidite, Cy5, and Cy5.5. BlackBerry quencher was attached to the 3’-end of TaqMan probes. The sequences of the primers and TaqMan probes are presented in Fig. S1. The PCR was performed with an initial step at 60°C for 1 min and a denaturation step at 95°C for 5 min, followed by 45–55 cycles at 95°C for 20 s and at 60°C for 60 s. Tissue samples of at least five individual bees were examined in triplicate. Mean copy numbers were calculated by using Rotor Gene Q software (Qiagen). Samples from each tissue were tested in at least two independent qPCR runs. Receptor transcript levels were normalized to elongation factor 1α (*Amef-1α*) transcript levels ( =  100%) by using the standard curve method. The standards covered copy numbers from 10^3^ – 10^7^. To detect statistically significant differences in gene expression, one-way ANOVA followed by Bonferroni’s multiple comparison test was applied on log(x + 1) transformed data as implemented in SPSS19 (Rel. 19.0.0, IBM SPSS Statistics).

### Construction of expression vectors

Expression-ready constructs of *Am5-ht2α* and Am5-*ht2αΔIII* were generated in pcDNA3.1 vector (Invitrogen). PCR was performed with specific primers (Table S1). PCR products were digested with *Nhe*I and *Eco*RI and subcloned. The resulting constructs were named pc*Am5-ht2α*-HA and pc*Am5-ht2αΔIII*-HA.

In order to generate an expression vector for *Am*5-*ht2β*, a PCR with specific primers (Fig. S1) was performed. The PCR product was digested with *Hind*III and *Age*I and subcloned into pcDNA6/*myc*-His A vector (Invitrogen) yielding pc*Am5-ht2β*-His. All insert fragments were checked by DNA sequencing.

### Functional expression of honeybee 5-HT_2_ receptors

Approximately 8 µg of pc*Am5-ht2α*-HA, pc*Am5-ht2αΔIII*-HA, and pc*Am5-ht2β*-His were transfected into exponentially growing HEK 293 cells (∼4×10^5^ cells per 5-cm Petri dish) by a modified calcium phosphate method [39]. Stably transfected cells were selected in the presence of the antibiotic G418 (0.8 mg/ml; pcDNA3.1 vector) or blasticidin (0.1 mg/ml; pcDNA6 vector). Isolated foci were propagated and analyzed for the expression of the receptor proteins either by immunocytochemistry and Western blotting (see Fig. S2) or by functional Ca^2+^ imaging upon receptor activation.

### Functional analysis of honeybee 5-HT_2_ receptors

The ability of the honeybee receptors to activate G_q_ proteins was assessed by monitoring changes in [Ca^2+^]_i_ with the Ca^2+^-sensitive fluorescent dye Fluo-4 (Invitrogen). Non-transfected HEK 293 cells and cells expressing Am5-HT_2α_, Am5-HT_2αΔIII_, or Am5-HT_2β_ were grown in 96-well plates to a density of ∼3×10^4^ per well. Cells were loaded at room temperature with Fluo-4 as described earlier [40]. Each 96-well plate was transferred into a fluorescence reader (FLUOstar Galaxy/Optima; BMG Labtech, Offenburg, Germany) to monitor Fluo-4 fluorescence. The excitation wavelength was 485 nm, and fluorescence emission was detected at 520 nm. Various concentrations of biogenic amines or receptor ligands were added, once Fluo-4 fluorescence had reached a stable value in each well. The changes in Fluo-4 fluorescence were recorded automatically. Dose-response curves for putative agonists/antagonists were established in at least two independent experiments.

## Results

### Cloning and sequence analysis of 5-HT_2_ receptors from *A. mellifera*

**Figure 1:**
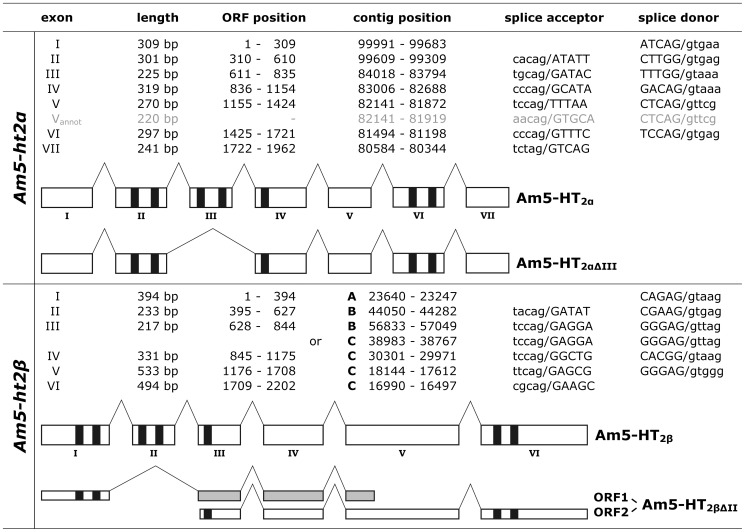
Genomic organization of *Am5-ht2α* and *Am5-ht2β* genes. The *Apis mellifera* genomic database (Amel_4.5) was screened with the *Am5-ht2α* and *Am5-ht2β* cDNA sequences. The figure shows the splicing pattern of the *Am5-ht2α* (upper part) and *Am5-ht2β* (lower part) genes. Nucleotide sequences of exons are given in upper-case letters and nucleotide sequences of introns are given in lower-case letters. Full-length and truncated receptor variants are displayed schematically. Boxes represent exons, and bent lines represent introns. The position of the seven trans-membrane domains is highlighted by black bars. Exons containing a frameshift are indicated in gray. The seven exons of the *Am5-ht2α* gene are completely covered in the genomic contig NW_001253562. The six exons of the *Am5-ht2β* gene are dispersed in three genomic contigs (A: NW_001262451, B: NW_001252966, und C: NW_001262048).

Two candidate genes encoding putative serotonin 5-HT_2_ receptors were identified in the completely sequenced honeybee genome (Am16 and Am17; [10]). Here, we used this sequence information and applied a PCR-based strategy to amplify the full-length cDNAs. The *Am5-ht*2*α* cDNA (Am16 in [10]) contains an open reading frame (ORF) of 1,962 bp and encodes a protein of 653 amino-acid residues (Am5-HT_2α_) with a calculated molecular weight of 71.5 kDa. The hydropathy profile and topology predictor *Phobius* [41] suggest seven trans-membrane (TM) domains, which are characteristic of GPCRs. The TM segments are flanked by an extracellular N-terminus of 140 and an intracellular C-terminus of 96 residues. An alignment of the cDNA with the genomic database (release Amel4.5) revealed that the *Am5-ht*2*α* gene contains seven exons and six introns (Fig. 1), and that it is located on chromosome LG9. *Am5-ht2α* is the ortholog of the *D. melanogaster 5-ht2α* gene (CG1056) with which it has three introns in common (Fig. S3A+C). A remarkable difference exists between the cloned cDNA and the annotated *Am5-ht*2*α* gene sequence (accession no.: XM394798). Exon V of the cDNA consists of 270 bp, whereas the annotated exon V contains only 220 bp (Fig. 1, V_annot_). This discrepancy originates from the usage of a different 5’ splice site during the *in silico* assembly of the gene, such that the annotated exon V starts at position 1,202 of the *Am5-ht*2*α* ORF. The 3’ splice sites of both the cloned and the annotated sequences are completely conserved (Fig. 1). Notably, in all PCR experiments performed, we never obtained a fragment as predicted by the annotation.

However, we isolated a splice variant of *Am5-ht2α* mRNA (*Am5-ht*2*αΔIII*). In *Am5-ht*2*αΔIII*, exon III (225 bp) is absent, which results in a shortened ORF of 1,737 bp (Fig. 1). This mRNA variant can potentially be translated into a protein of 578 amino acid residues. Compared with the full-length receptor, this protein will lack TM3, TM4, and the entire CPL2 (see Fig. 1).

In addition to *Am5-ht2α*, three partial mRNAs that might encode proteins have been annotated *in silico*. However, with 256 bp (XM_001119970), 522 bp (XM_001122856), and 1,053 bp (XM_624894), these sequences are too short to code for complete GPCRs. Nevertheless, the deduced amino-acid sequences showed striking sequence similarity to a 5-HT receptor of the spiny lobster, *Panulirus interruptus* (Pan5-HT_2β_; [7]; accession no.: AY550910). We used the crustacean sequence to design primers annealing in close proximity to the start and the stop codons of the hypothetical honeybee ortholog. A single cDNA, which contained an ORF of 2,202 bp, was amplified on honeybee brain cDNA (*Am5-ht2β*). The ORF is distributed on three genomic contigs (Fig. 1). Contig NW_001252966 has been allocated to chromosome LG10, whereas the other two, i.e., NW_001262451 and NW_001262048, have not been assigned to any chromosome, as yet. The coding sequence of the gene is interrupted by five introns (Fig. 1) with two of them being in common with the orthologous gene in *D. melanogaster* (CG42796, Fig. S3B+C). The ORF of the *Am5-ht2β* gene encodes a protein (Am5-HT_2β_) of 733 amino-acid residues with a calculated molecular weight of 80.7 kDa. The Am5-HT_2β_ receptor contains an extremely long CPL3 of 399 residues and a relatively short C-terminus of 25 residues.

Similar to *Am5-ht*2*α*, we identified a splice variant of the *Am5-ht2β* mRNA. In this variant (*Am5-ht2βΔII*), exon II is completely missing. This results in a frameshift of the ORF (ORF1) with an early stop codon at position 1,075 - 1,077 (Fig. 1). ORF1 is predicted to code for a protein of 358 amino-acid residues and a calculated molecular weight of 39.5 kDa. The sequence from Met_1_ to Gly_132_ including TM1, TM2, and the extracellular loop (ECL) 1 is identical to the full-length Am5-HT_2β_ protein. Because of the frameshift, the consecutive sequence (Gly_133_ to Pro_357_) differs completely from the full-length receptor protein. A second ORF (ORF2, 1,605 bp) starts within exon III and lasts to the stop codon of the full-length mRNA. This reading frame is predicted to encode a protein of 491 residues with a calculated molecular weight of 54.1 kDa. The amino-acid sequence is completely identical to that of full-length Am-5-HT_2β_ from Met_243_ (ECL2 prior to TM5) to Arg_733_ but obviously misses a large part of the N-terminal region encompassing TM domains 1-4 of an intact GPCR (Fig. 1).

**Figure 2:**
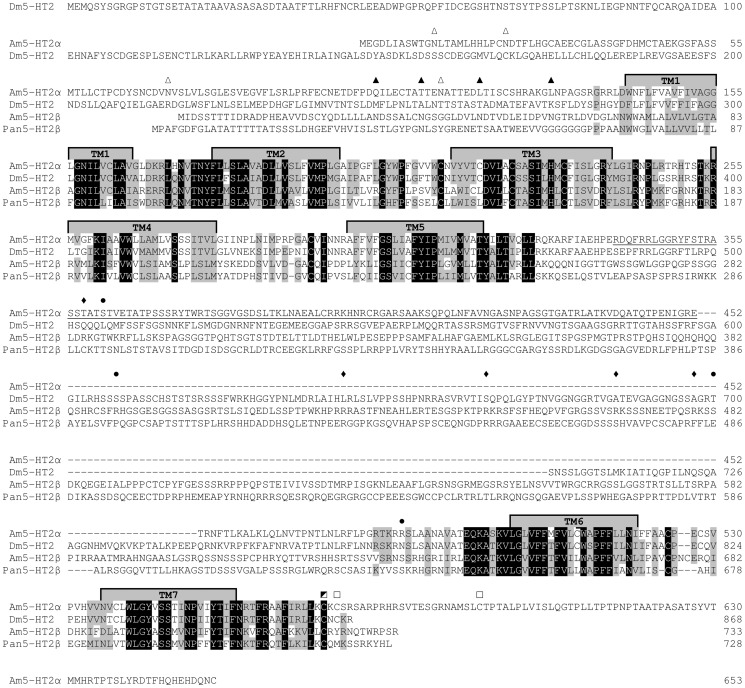
Amino-acid sequence alignment of Am5-HT_2α_, Am5-HT_2β_, and orthologous receptors from *Drosophila melanogaster* (Dm5-HT_2_; accession no. CAA57429) and *Panulirus interruptus* (Pan5-HT_2β;_ AAS57919). Identical residues between all four receptors are shown as white letters against black, whereas conservatively substituted residues are shaded. Putative trans-membrane domains (TM1–7) are indicated by gray bars. Putative consensus sites for post-translational modifications in Am5-HT_2α_ and Am5-HT_2β_ are indicated by open and filled symbols, respectively: N-glycosylation sites are shown as triangles, PKA phosphorylation sites as diamonds, PKC phosphorylation sites as circles, palmitoylation sites as squared boxes. Underlined letters represent the region within the CPL3 of Am5-HT_2α_ used to raise specific antibodies (see Fig. S2). The amino acid position is indicated on the right.

Sequence motifs, which are essential for the three-dimensional structure, ligand binding, and signal transduction of the receptor, are well conserved in both full-length Am5-HT_2α_ and Am5-HT_2β_ proteins (Fig. 2). An exception is the Asp residue within the D-R-Y motif required for G protein coupling [42]; this is substituted by Gly in Am5-HT_2α_ (G_237_-R_238_-Y_239_, Fig. 2). Five and four consensus motifs for potential N-glycosylation (N-x-[S/T]) are located in the extracellular N-terminus of Am5-HT_2α_ and Am5-HT_2β_, respectively (Fig. 2). Cysteine residues in the C-terminus (Cys_570_, Cys_572_, and Cys_594_ in Am5-HT_2α_, Cys_722_ in Am5-HT_2β_, Fig. 2) are possible sites for post-translational palmitoylation. Whereas no consensus site for phosphorylation by protein kinase A (PKA, R-[R/K]-x-[S/T]) or protein kinase C (PKC, [S/T]-x-[R/K]) is found in Am5-HT_2α_, five PKA consensus sites and four PKC consensus sites are present within CPL3 of Am5-HT_2β_ (Fig. 2).

**Figure 3:**
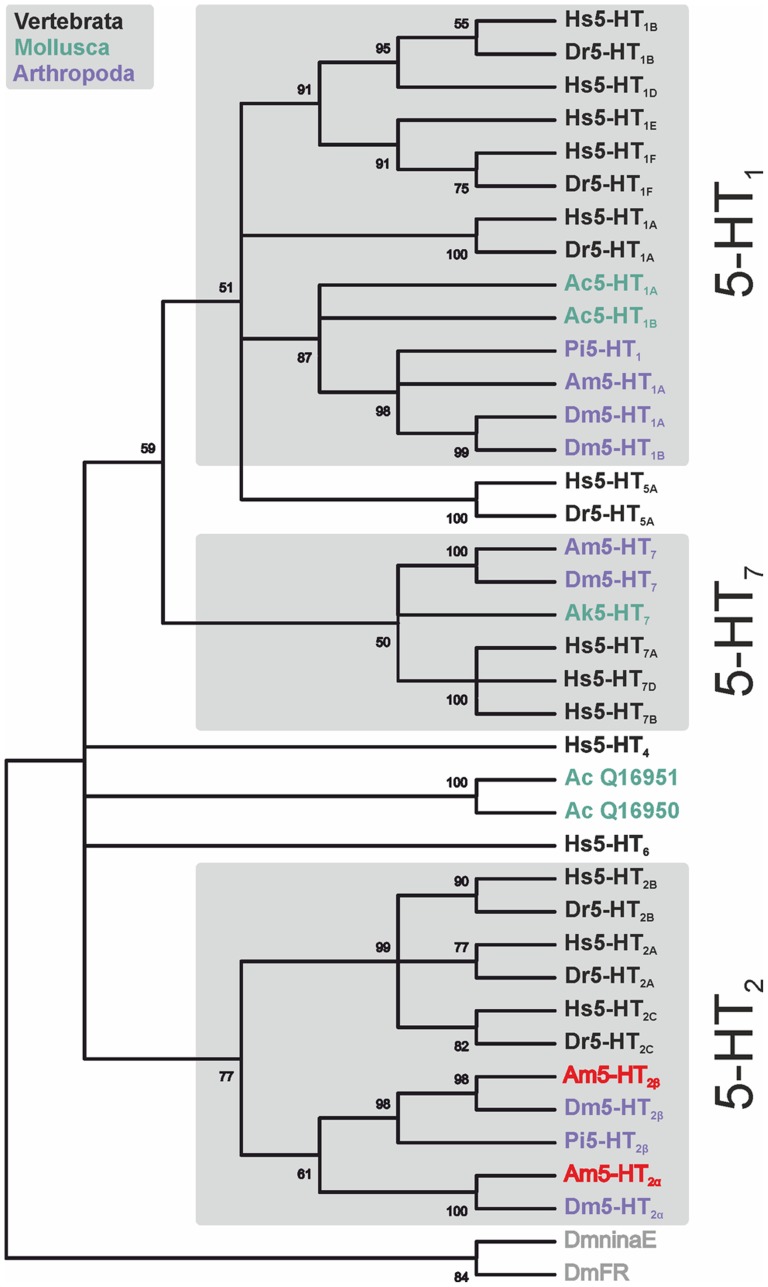
Phylogenetic relationship between various 5-HT receptors from vertebrates, mollusks, and arthropods. Alignments were performed with BioEdit (version 7.0.5) by using the core amino-acid sequences lacking the variable regions of the N- and C-terminus and the third cytoplasmic loop. Maximum parsimony analysis was calculated with MEGA4. The receptor sequences followed by their accession numbers are listed in the order illustrated: *Homo sapiens* (Hs5-HT_1B_, acc. no. NP_000854), *Danio rerio* (Dr5-HT_1B_, AAI63698), Hs5-HT_1D_ (NP_000855), Hs5-HT_1E_ (NP_000856), Hs5-HT_1F_ (NP_000857), Dr5-HT_1F_ (NP_001076425), Hs5-HT_1A_ (NP_000515), Dr5-HT_1A_ (NP_001139238), *Aplysia californica* (Ac5-HT_1_, AAM46088, AAC28786), *Panulirus interruptus* (Pi5-HT_1_, AY528822), *Apis mellifera* (Am5-HT_1_, CBI75449), *Drosophila melanogaster* (Dm5-HT_1A_, CAA77570), Dm5-HT_1B_ (CAA77571), Hs5-HT_5A_ (NP_076917), Dr5-HT_5A_, (NP_001119882), Am5-HT_7_ (AM076717), Dm5-HT_7_ (NP_524599), *Aplysia kurodai* (Ak5-HT_7_, ACQ90247), Hs5-HT_7A_ (NP_000863), Hs5-HT_7D_ (NP_062873), Hs5-HT_7B_ (NP_062874), Hs5-HT_4_ (NP_001035259), Ac5-HT_2_ (Q16951), Ac5-HT_1_ (Q16950), Hs5-HT_6_ (NP_000862), Hs5-HT_2B_ (NP_000858), Dr5-HT_2B_ (ABI18978), Hs5-HT_2A_ (NP_000612), Dr5-HT_2A_ (CAQ15355), Hs5-HT_2C_ (NP_000859), Dr5-HT_2C_ (CAX14715), Am5-HT_2β_ (FR727108), Dm5-HT_2β_ (NM_141548/NM_169229), Pi5-HT_2β_ (AY550910), Am5-HT_2α_ (FR727107), and Dm5-HT_2α_ (CAA57429). The divergent *D. melanogaster* ninaE-encoded rhodopsin 1 (DmninaE, NM_079683) and *D. melanogaster* FMRFamide receptor (DmFR, AAF47700) were used as out-groups. The numbers at the nodes of the branches represent the percentage bootstrap support (2,000 replications) for each branch.

A comparison of Am5-HT_2α_ and Am5-HT_2β_ amino-acid sequences with NCBI databases identified several orthologous protostomian and deuterostomian 5-HT_2_ receptors. For Am5-HT_2α_, the highest amino acid identity/similarity (ID/S) existed to the 5-HT_2_ receptor of *D. melanogaster* (Dm5-HT_2α_; [13]; ID 28.4%, S 34.8%). For Am5-HT_2β_, homology was more pronounced to 5-HT_2_ receptors from the crustaceans *Panulirus interruptus* (Pan5-HT_2β_; [7]; ID 31.2%, S 42.8%) and *Procambarus clarkii* (Pro5-HT_2_; [8]; ID 33.2%, S 40.4%). In phylogenetic tree analyses, both Am5-HT_2α_ and Am5-HT_2β_ always grouped with protostomian and deuterostomian 5-HT_2_ receptors (Fig. 3).

### Tissue-specific expression patterns of the Am5-HT_2_ genes

**Figure 4:**
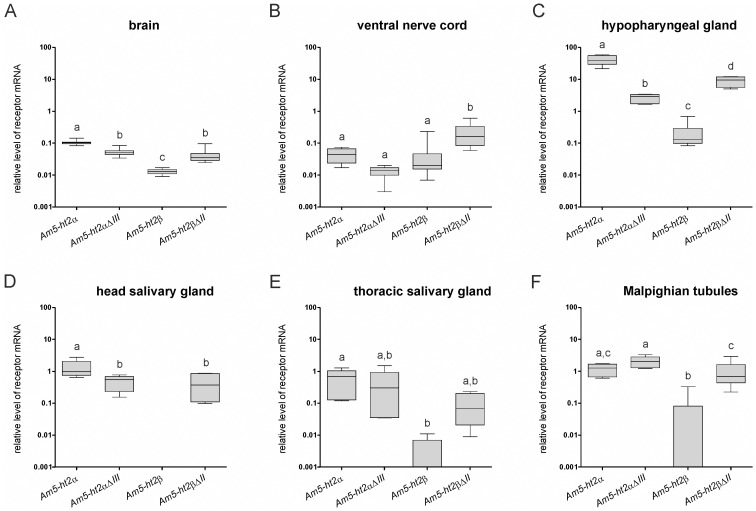
Tissue-specific expression patterns of *Am5-ht2* receptor genes in pollen foragers determined by quantitative real-time PCR. Transcript levels were normalized to *Amef-1α*. (A) Relative levels of receptor-gene mRNAs in the brain (n = 11). (B) Relative levels of receptor-gene mRNAs in the ventral nerve cord (*Am5-ht2α* and *Am5-ht2αΔIII*: n = 11; *Am5-ht2β* and *Am5-ht2βΔII*: n = 10) (C) Relative levels of receptor-gene mRNAs in the hypopharyngeal gland (*Am5-ht2α*: n = 6; *Am5-ht2αΔIII*: n = 5; *Am5-ht2β*: n = 7; *Am5-ht2βΔII*: n = 6). (D) Relative levels of receptor-gene mRNAs in the head salivary gland (*Am5-ht2α* and *Am5-ht2αΔIII*: n = 5; *Am5-ht2β* and *Am5-ht2βΔII*: n = 7). (E) Relative levels of receptor-gene mRNAs in the thoracic salivary gland (each: n = 5). (F) Relative levels of receptor-gene mRNAs in Malpighian tubules (*Am5-ht2α*: n = 5; *Am5-ht2αΔIII*: n = 6; *Am5 ht2β*: n = 8; *Am5-ht2βΔII*: n = 7). Groups that differed significantly from one another in relative mRNA levels are indicated with different letters above the box plots (p < 0.05, Bonferroni’s multiple comparison test).

The tissue-specific distribution of *Am5-ht2α* and *Am5-ht2β* mRNA was determined by qPCR in tissue samples from pollen foragers and drones (Fig. 4, Fig. S4). Application of splice-variant-specific primers (Fig. S1) allowed us to unravel the expression pattern of the different splice variants for each receptor gene. PCR fragments originating from full-length and splice variants of *Am5-ht2α* and *Am5-ht2β* were found in the CNS, the hypopharyngeal glands, the salivary glands, and the Malpighian tubules of pollen foragers (Fig. 4; see Fig. S4 for drone tissues). Expression of *Am5-ht2α* was higher than *Am5-ht2β* expression, e.g., up to ten times in the nervous system and ∼200 times in the hypopharyngeal glands. Interestingly, in the brain and in the hypopharyngeal gland, the expression of the *Am5-ht2αΔIII* transcript was lower than the full-length version, whereas the opposite held true for the *Am5-ht2β* and *Am5-ht2βΔII* transcripts (Fig. 4). All transcripts showed similar expression patterns in the CNS of drones compared with female honeybees (Fig. 4A+B; Fig. S4).

### Functional analyses of Am5-HT_2_ receptors in HEK 293 cells

**Figure 5:**
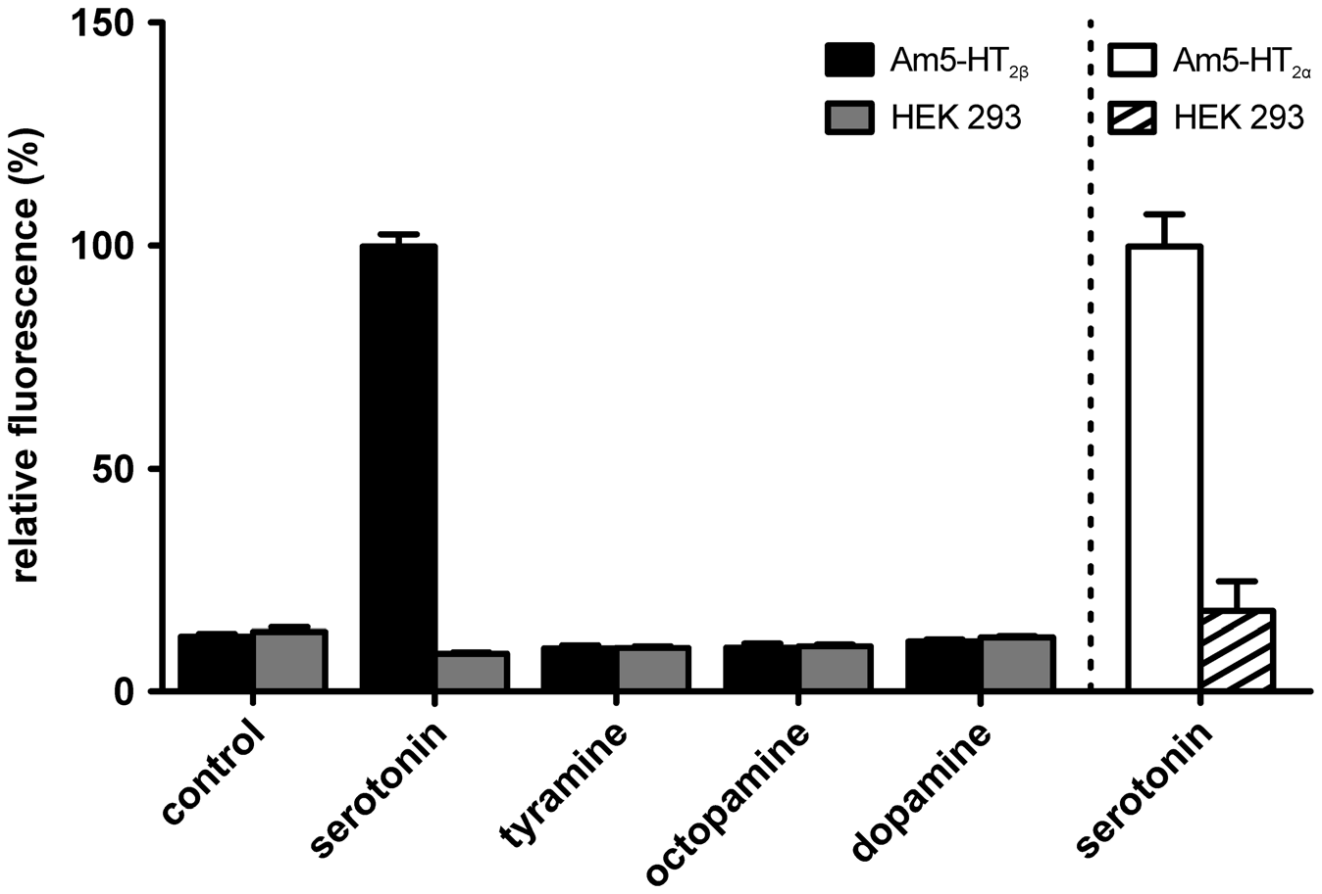
Biogenic amine (10 µM each) modulation of intracellular Ca^2+^ concentrations in HEK 293 cells constitutively expressing Am5-HT_2α_ or Am5-HT_2ß_ receptors and in non-transfected HEK 293 cells. Cells were loaded with the Ca^2+^-sensitive dye Fluo-4 and changes in [Ca^2+^]_i_ were measured fluorimetrically. All values of HEK 293 cells stably expressing Am5-HT_2β_ (black bars) and the corresponding non-transfected HEK 293 cells (gray bars) were normalized to the serotonin response of the Am5-HT_2β_-expressing cell line. The values obtained for the Am5-HT_2α_ cell line (white bar) and non-transfected HEK 293 cells (striped bar) were normalized analogously. Data represent the mean ± SD of octuplicate determinations from a representative experiment. The values for serotonin-stimulated Am5-HT_2α_- and Am5-HT_2β_-expressing cells are significantly different from all other values (one-way ANOVA, followed by Dunnett’s multiple comparison test, p < 0.05).

5-HT_2_ receptors are known to induce an increase in [Ca^2+^]_i_. We generated HEK 293 cell lines constitutively expressing either Am5-HT_2α_ or Am5-HT_2β_ to examine whether these honeybee receptors also induced serotonin-dependent Ca^2+^ signals. In a first series of experiments, we analyzed the ligand specificity of both receptors. Only serotonin (10 µM) increased [Ca^2+^]_i_ in Am5-HT_2α_- and Am5-HT_2β_-expressing cells, whereas no signals were observed after the application of 10 µM tyramine, octopamine, or dopamine (Fig. 5). Non-transfected cells did not respond to any of these biogenic amines (Fig. 5). A third cell line expressing the alternatively spliced variant Am5-HT_2αΔIII_ did not respond to the application of any biogenic amine, including serotonin. Therefore, this construct most likely does not code for a *bona fide* GPCR.

**Figure 6:**
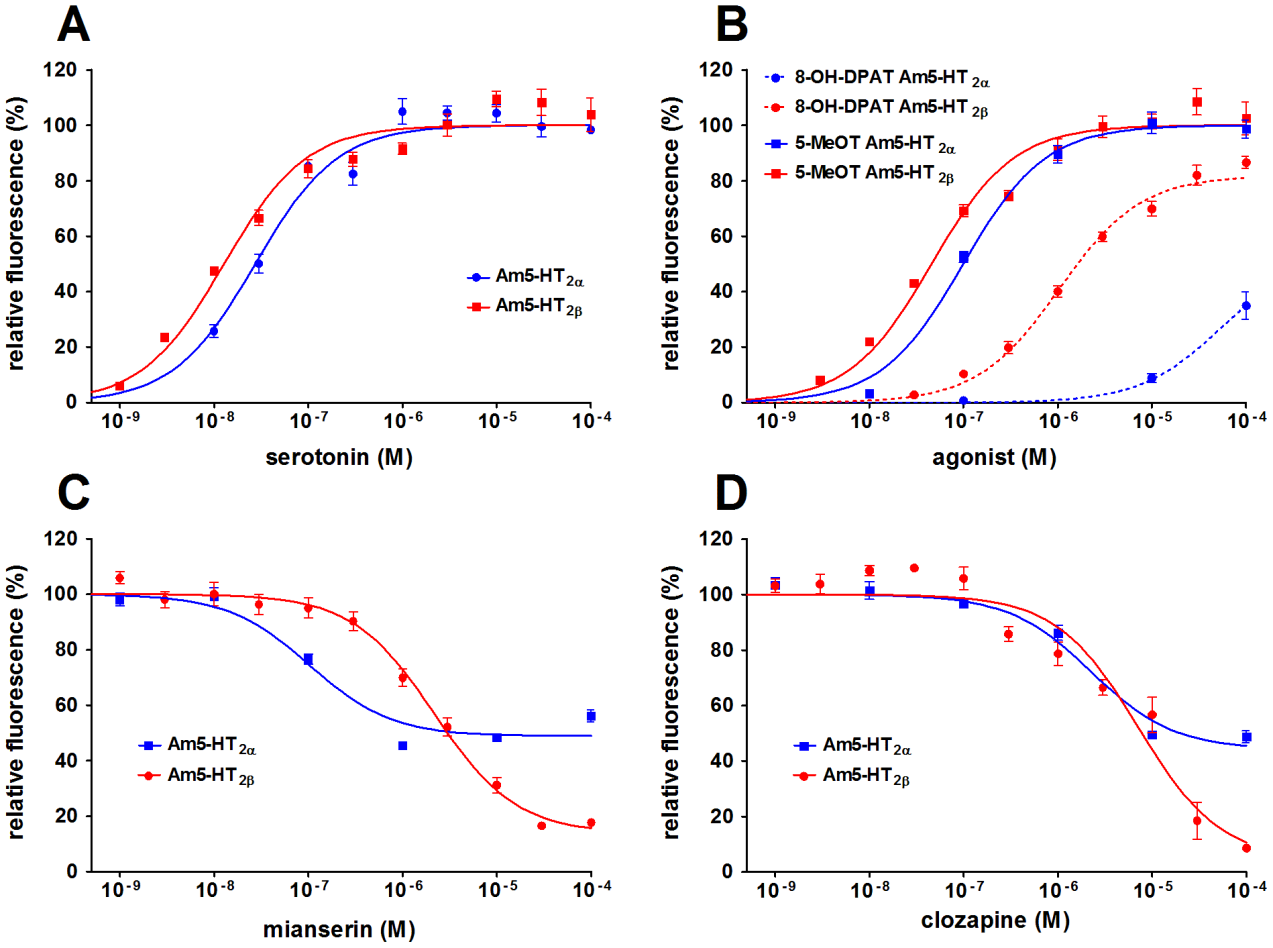
Concentration-dependent effects of agonists and antagonists on [Ca^2+^]_i_ in Am5-HT_2α_- and Am5-HT_2β_-expressing HEK 293 cells. [Ca^2+^]_i_ is depicted as relative fluorescence given in percent. Data represent the mean ± SEM of eight replicates from one experiment representative of at least two similar experiments. (A) Am5-HT_2α_- or Am5-HT_2β_-expressing cells were incubated with increasing concentrations of serotonin. Values were normalized to the response measured at the highest serotonin concentration ( = 100%) for each cell line. (B) Cells expressing Am5-HT_2α_ or Am5-HT_2β_ were incubated with increasing concentrations of the agonists 5-methoxytryptamine (5-MeOT) and 8-hydroxy-DPAT (8-OH-DPAT). Values were normalized to the response measured at the highest 5-MeOT concentration ( = 100%) for each cell line. (C, D) Cells expressing Am5-HT_2α_ or Am5-HT_2β_ were incubated with 50 nM serotonin and increasing concentrations of the antagonist mianserin (C) or clozapine (D). Values were normalized to the serotonin response in the absence of antagonist ( = 100%).

For both full-length receptors, the concentration-response relationship for serotonin was examined with concentrations ranging from 1 nM to 300 µM. In cell lines expressing either Am5-HT_2α_ or Am5-HT_2β_, the serotonin effect was concentration-dependent and saturable, resulting in sigmoidal concentration-response curves (Fig. 6A). The half-maximal effective concentrations (EC_50_) were similar at 25.7 nM and 32.5 nM for Am5-HT_2α_ and Am5-HT_2β_, respectively. Maximal increase in [Ca^2+^]_i_ was observed at serotonin concentrations of ≥1 µM.

To establish pharmacological profiles for both 5-HT_2_ receptors, the effects of various 5-HT receptor agonists and antagonists were tested. Two agonists, namely 5-methoxytryptamine and 8-hydroxy-2-(di-n-propylamino)tetralin (8-OH-DPAT), caused an increase in [Ca^2+^]_i_ in both receptor-expressing cell lines (Fig. 6B). At the Am5-HT_2α_ receptor, the EC_50_ values were 70 nM and 55.9 µM for 5-methoxytryptamine and 8-OH-DPAT, respectively. At the Am5-HT_2β_ receptor, the EC_50_ for 5-methoxytryptamine was 60.4 nM, and for 8-OH-DPAT, the EC_50_ was 561.5 nM. Neither 5-methoxytryptamine nor 8-OH-DPAT had an effect on non-transfected HEK 293 cells. In contrast to the previously mentioned substances, 5carboxamidotryptamine and 2,5-dimethoxy-4-iodoamphetamine even affected [Ca^2+^]_i_ in non-transfected cells and thus were not examined with the receptor-expressing cell lines.

The potency of 5-HT-receptor antagonists to inhibit serotonin-induced Ca^2+^ responses in Am5-HT_2α_- and Am5-HT_2β_-expressing cells was examined in another series of experiments. The calculated IC_50_ and maximal inhibitory effect (I_max_) of the substances are summarized in Table 1, and examples of concentration-response relationships for mianserin and clozapine are shown in Figures 6C+D. Most antagonists were able to inhibit the activation of the Am5-HT_2α_ receptor by 30-60%. At the Am5-HT_2α_ receptor, antagonists showed decreasing inhibitory potency with: SB200646  =  mianserin > cyproheptadine > methiothepin > clozapine > methysergide.

**Table 1:** Antagonist profile of *Apis mellifera* 5-HT_2_ receptors.

antagonist	Am5-HT_2α_IC_50_ [µM]	Am5-HT_2α_maximal inhibition	Am5-HT_2β_IC_50_ [µM]	Am5-HT_2β_maximal inhibition
clozapine	1.7	56%	6.1	95%
cyproheptadine	0.7	64%	0.2	77%
ketanserin	-	no effect	1.5	89%
methiothepin	0.6	61%	-	no effect
methysergide	2.2	45%	-	no effect
mianserin	0.2	51%	2.4	76%
SB-200646	0.1	32%	-	no effect

IC_50_ values (potency) and relative efficacy were calculated from concentration-response curves for each drug. Efficacy is given as the maximal inhibition (%) of fluorescence compared with 50 nM serotonin in the absence of antagonist and calculated by curve fitting. Values are means of the results of two independent experiments, each performed in octuplicates.

Interstingly, SB-200646, methiothepin, and methysergide showed no effects at the Am5-HT_2β_ receptor, whereas clozapine, cyproheptadine, ketanserin, and mianserin were strongly inhibitory with a maximal inhibitory effect of ≥77%. In comparison with Am5-HT_2α_, the rank order of inhibitory potency at Am5-HT_2β_ was different with: cyproheptadine > ketanserin > mianserin > clozapine.

## Discussion

The intense arborization of serotonergic neurons in the honeybee CNS suggests that serotonin controls the activity of many neuronal circuits [11]. In order to obtain further insight into serotonergic signaling in the honeybee, the molecular and functional identity of the 5-HT receptor repertoire of the honeybee has to be unraveled. Concomitantly with the release of the honeybee genomic sequence [37], several 5-HT receptor genes were annotated [10]. These *in silico* analyses, however, lack experimental data related to pharmacological properties, expression patterns, and physiological functions of the identified receptors. So far, only a 5-HT_1A_ receptor [32] and a 5-HT_7_ receptor [33] have been thoroughly characterized. With the analysis of the two paralogous 5-HT_2_ receptors, Am5-HT_2α_ and Am5-HT_2β_, the current work provides an important step to complement our knowledge of the serotonergic receptor system in the honeybee.

### Genomic organization and gene prediction

The coding sequences of *Am5-ht2α* and *Am5-ht2β* are heavily spliced and consist of seven and six exons, respectively. Two splice sites are located in similar positions, one in ECL1 and a second in ECL2. These two splice sites are not only conserved in orthologous genes of the fruit fly (Fig. S3) but also represent the positions from where the splice variants originate (Fig. 1).

A comparison of cloned and annotated *Am5-ht2*-receptor gene sequences reveals several discrepancies. Whereas the overall exon-intron structure is similar, the annotated *Am5-ht2α* sequence contains a shorter exon V (Fig. 1). Obviously, native honeybee spliceosomes not only use exactly conserved splice donor and acceptor sites, which, however, are central elements in gene prediction algorithms [43]. The situation for the *Am5-ht2β* gene is even more complicated. Alignment of the cloned cDNA with the honeybee genome sequence showed that: (1) the *Am5-ht2β* gene contains extremely large introns (>100,000 bp), which are hardly recognized by gene prediction software [44]; (2) the *Am5-ht2β* gene is dispersed over three genomic contigs; (3) the ORF of *Am5-ht2β* is oriented in either a 5’ to 3’ or 3’ to 5’ orientation compared with deposited contig sequences. This obviously prevents a computer-based assembly of the correct ORF. As exemplified by *Am5-ht2β*, gene annotations provide important information, but molecular cloning of cDNA remains a prerequisite to determine the functional properties of a protein.

### Phylogenetic analysis of 5-HT_2_ receptors

Phylogenetic analyses of various 5-HT receptors revealed a separation of two main groups (Fig. 3). One group comprises receptors coupled to the cAMP pathway, i.e., 5-HT_1_ (G_i_-coupled) and 5-HT_7_ receptors (G_s_-coupled). The other group comprises 5-HT_2_ receptors preferentially coupled to G_q_ proteins. Interestingly, deuterostomian and protostomian 5-HT receptors do not form distinct branches suggesting common ancestors for each receptor class. In arthropods, usually one or two receptor subtypes exist per 5-HT receptor class. In *D. melanogaster*, the Dm5-HT_1A_ and Dm5-HT_1B_ receptors result from a recent duplication event [12]. So far, one 5-HT_1_ receptor has been characterized in the honeybee (Am5-HT_1A_, [32]). In addition, one 5-HT_7_ receptor has been found in *D. melanogaster* and *A. mellifera* [14], [33]. Here, we have added two functional 5-HT_2_ receptors to the list of honeybee 5-HT receptors. A receptor orthologous to Am5-HT_2α_ has been characterized in *D. melanogaster* (CG1056; [13]). The *D. melanogaster* genome contains another gene (CG42796) potentially encoding an ortholog of Am5-HT_2β_. This hypothesis, however, remains to be established experimentally.

### Am5-ht2 genes give rise to several transcripts by alternative splicing

Despite the apparent functional diversity afforded by the existence of multiple 5-HT_2_ receptor subtypes, the existence of functional splice variants of these receptors adds to the potential repertoire of 5-HT receptors an additional mode of regulation. This phenomenon was observed for several 5-HT_2_ receptor subtypes (*Ascaris suum* 5-HT_2_: [45]; 5-HT_2A_: [46]; 5-HT_2C_: [47], [48]). Splice variants differ in distribution, ligand-binding properties and functional signaling (for a review, see: [49]).

For both honeybee 5-HT_2_ receptor genes, only one splice product contains information for a complete GPCR (Fig. 1). Shorter versions are generated by exon skipping. Interestingly, *Am5-ht2αΔIII* and *Am5-ht2βΔII* have lost the exon coding for a central part of the receptor including TM3 and TM4 (Fig. 1). Several 5-HT_2_ receptor splice variants affecting the same region were also reported in mammalian genes [46], [47]. A deletion covering the region encoding CPL2 and TM4 leads to a frameshift in the downstream sequence and a premature stop codon of the rat 5-HT_2C_ receptor [47]. Similarly truncated forms were also detected in cDNA samples from mouse and human brain [47]. A variant of the human 5-HT_2A_ receptor contains an 118 bp insertion which produces a frameshift and a premature stop codon [46]. Unfortunately, nothing is known about the function(s) of truncated 5-HT_2_ receptors in mammals.

When heterologously expressed, the Am5-HT_2αΔIII_ receptor is synthesized as a glycosylated and non-glycosylated protein suggesting that it is processed in the Golgi apparatus (Fig. S2A). Attempts to monitor functional receptor activity from this construct were unsuccessful. This might be attributable to the absence, in TM3, of critical residues that interact with the amino group of biogenic amines [50]. Although the protein is not functional on its own, it might assemble with a full-length receptor and thereby modulate the binding and/or signal transduction properties in a heterodimeric complex. Such modes of regulation have been observed for a *Caenorhabditis elegans* dopamine receptor variant lacking TM6 and TM7 [51] and for mutants of the human dopamine D2 receptor [52].

Deletion of exon II in *Am5-ht2βΔII* leads to a frameshift in the ORF, which is terminated by an early stop codon. Thus, ORF1 of *Am5-ht2βΔII* codes for a protein that shares, with full-length Am5-HT_2β_, the N-terminal part down to ECL1 but that thereafter contains a completely divergent sequence. A second ORF of this splice variant codes for a protein identical to the full-length receptor from TM5 down to the C-terminus (Fig. 1). Whether any of these proteins can be heterologously expressed and eventually modify the properties of the full-length receptor remains to be examined in a forthcoming study.

### Functional characterization of Am5-HT_2_ receptors

Am5-HT_2α_ and Am5-HT_2β_ were functionally expressed in HEK 293 cells. Like their mammalian orthologs, both receptors couple to the G_q_ signaling pathway. These results support the concept that the signaling pathway is conserved for a given receptor class, whereas pharmacological profiles might differ.

With EC_50_ values of 25.7 nM and 32.5 nM, respectively, Am5-HT_2α_ and Am5-HT_2β_ displayed similar potencies for serotonin as those also reported for 5-HT_2α_ receptors from *D. melanogaster* (Dm5-HT_2α_, 16 nM; [13]) and *Calliphora vicina* (Cv5-HT_2α_, 24 nM; [36]). In addition to serotonin, 5-methoxytryptamine also activates honeybee 5-HT_2_ receptors but with 2-fold higher EC_50_ values (Fig. 6B). In the blowfly *C. vicina*, nanomolar concentrations of 5-methoxytryptamine activate only Cv5-HT_2α_ (EC_50_ = 67 nM) but not Cv5-HT_7_ [36]. However, at higher concentrations (EC_50_ = 55.9 µM and 560 nM, respectively), both honeybee receptors are also activated by 8-OH-DPAT, as is Cv5-HT_2α_ (EC_50_ = 62 µM). In conclusion, both 5-methoxytryptamine and 8-OH-DPAT can be considered as non-selective 5-HT receptor agonists in the honeybee, because 5methoxytryptamine also activates the Am5-HT_1A_ receptor [32], and because 8-OH-DPAT is a poor agonist at the Am5-HT_7_ receptor [33].

In general, the pharmacological profiles of both Am5-HT_2_ receptors are different for antagonists, especially for SB-200646, methiothepin, and methysergide, which display high potency at Am5-HT_2α_ but do not block Am5-HT_2β_. To be considered as an Am5-HT_2α_-specific antagonist, however, these substances need to be re-examined at the Am5-HT_1_ and Am5-HT_7_ receptors. Clozapine displays similar potencies for both 5-HT_2_ receptors. In contrast, mianserin has a 24fold higher potency at the Am5-HT_2α_ receptor (Table 1) and thus is a promising candidate for an Am5-HT_2α_–specific antagonist. Interestingly, ketanserin seems to be a specific antagonist for Am5-HT_2β_. However, these substances also remain to be tested with Am5-HT_1_ and Am5-HT_7_.

We have demonstrated that many drugs are active at several 5-HT receptor subtypes of the honeybee ([32], [33]; this study). Thus, when applying an agonist to elicit a physiological or behavioral “serotonin effect”, the possible activation of multiple 5-HT receptors or even other aminergic receptors simultaneously should not be overlooked.

### Localization of Am5-HT_2_ receptors

The expression patterns of only two 5-HT receptors have been reported in the honeybee. The Am5-HT_1A_ receptor is restricted to neuronal tissues [32]. Transcripts of Am5-HT_7_ have been detected in neural and peripheral tissues [33]. Based on our qPCR data, both *Am5-ht2* receptor genes share the widespread expression pattern with *Am5-ht7*.

Transcript levels for *Am5-ht2α* were clearly higher than those for *Am5-ht2β*, especially in the hypopharyngeal gland (Fig. 4C). The hypopharyngeal gland plays an important role in the control of eusocial behavior and displays ‘functional flexibility’ in secreting either royal jelly compounds or digestive enzymes depending on the age and task of the worker bee [53], [54]. Queen caste development strongly depends on royal jelly [55], [56]. A tempting speculation is that glandular activity is modulated by serotonin, and thus, signaling *via* Am5-HT_2α_ is crucial for generating the female reproductive caste in honeybees.

The *Am5-ht2β* gene is expressed in the nervous system of worker bees and drones. In *D. melanogaster*, the orthologous receptor (CG42796) is preferentially expressed in the male CNS [57]. Thus, 5-HT_2β_ receptors might be involved in different functional circuits in these insects.

With the present work, we extend the basic knowledge of the serotonergic system of the honeybee by adding two receptors, Am5-HT_2α_ und Am5-HT_2β_, to the growing list of well-characterized honeybee GPCRs. The detailed knowledge of the pharmacological properties of a receptor is essential for designing and conducting targeted pharmacological experiments (e.g., [32]). In combination with expression data, knowledge of the pharmacological profiles of these receptors should now facilitate such studies for individual 5-HT receptor subtypes in the honeybee.

## Supporting Information

Figure S1 **Sequences of primers and TaqMan probes (including 5’- and 3’-modifications; see material and methods) used for qPCR assays and the expected length of the resulting amplicons.** The positions of primers on the cDNAs are schematically shown by open arrows, TaqMan probes are indicated as asterisks.
(PDF)

Figure S2 **Western blot and immunocytochemical analyses of Am5-HT_2α_- and Am5-HT_2αΔIII_-expressing cell lines.** Anti-Am5-HT_2α_ antibodies were raised against a fusion protein containing part of the third cytoplasmic loop (CPL3; amino acid Arg_340_ to Glu_452_; see Fig. 1). The cDNA fragment was amplified by PCR with specific primers (Table S1). The fragment was cloned into pMAL-c2X vector (New England Biolabs, Frankfurt, Germany). The fusion protein was over-expressed in *E. coli* BL21 CP and purified by amylose affinity-chromatography (New England Biolabs). In collaboration with the Nachwuchsgruppe Antikörper-Technologien (University of Potsdam, Germany), the fusion protein was used to immunize mice and to raise monoclonal antibodies. A second fusion protein containing a His-tag attached to the same receptor fragment was expressed from pET-30a vector (Novagen, Darmstadt, Germany) and used for testing the specificity of the monoclonal antibodies. Membrane proteins (10 µg protein per lane) of human embryonic kidney cells (HEK 293) expressing Am5-HT_2α_-HA and Am5-HT_2αΔIII_-HA receptors (see below) were isolated as previously described (Thamm et al., 2010). Proteins were separated by SDS polyacrylamide gel electrophoresis on 10% or 12% gels and transferred to polyvinylidene fluoride membranes (Roth, Karlsruhe, Germany). These membranes were blocked with 5% (w/v) dry milk in Tris-buffered saline containing Tween 20 (TBS-T, 10 mM Tris-HCl, pH 7.5, 150 mM NaCl, 0.01% Tween 20) for 30 min at room temperature, incubated either with specific anti-HA antibodies (Anti-HA High Affinity, Roche, Penzberg, Germany; dilution 1∶5,000) or with receptor-specific antibodies (dilution 1∶100) in TBS-T, washed with TBS-T, and finally incubated with secondary antibodies (1∶5,000, anti-rat-HRP; American Qualex, La Mirada, USA; 1∶200, anti-mouse Alexa568; Invitrogen) for 1 h. Signals were visualized by enhanced chemiluminescence. (**A**) Western blot analyses of membrane proteins (10 µg per lane) of non-transfected HEK 293 cells (nt) and HEK 293 cells expressing either full-length Am5-HT_2α_-HA (full) or Am5-HT_2αΔIII_-HA (ΔIII) proteins. Both anti-HA (dilution 1:5,000, left) and anti-Am5-HT_2α_ (culture supernatant 1∶10, right) antibodies recognize bands of identical size in protein preparations from transfected cells. No bands were detected in protein preparations from non-transfected cells. (**B**) Similar staining patterns were observed in receptor-expressing cell lines with both antibodies (anti-HA and anti-Am5-HT_2α_). Scale bar 40 µm). Unfortunately, the anti-Am5-HT_2α_ antibody did not work with native tissue, neither on Western Blots nor on fixed tissue sections. This is probably due to the very low endogenous expression level of Am5-HT_2α_, especially in nervous tissue.
(PDF)

Figure S3 **Comparison of splice sites in** ***5-ht2*** **genes (coding regions) of** ***Apis mellifera*** **and** ***Drosophila melanogaster*** **.** Splice donor and acceptor sites were identified using the Splign alignment tool (http://www.ncbi.nlm.nih.gov/sutils/splign/splign.cgi). In the mRNA sequence alignments (**A+B**), which were created using the program BioEdit version 7.1.3.0, splice sites are indicated by +++ and highlighted in yellow and green for *5-ht2* genes of the honeybee and *D. melanogaster*, respectively. (**A**) *Am5-ht2α* is the orthologue of the *Dm5-ht2α* gene (CG1056) with which it has three introns in common. (**B**) *Am5-ht2β* is the orthologue of the *D. melanogaster* CG42796 gene with which it has two introns in common. (**C**) A pictogram of a GPCR with its seven transmembrane segments (green bars) is displayed. Arrows point to the relative positions of splice sites in the primary structures of the receptors. For each exon, the last amino acid residue is indicated and numbered according to its position in the deduced amino acid sequence. One splice site is conserved in all four *5-ht2* genes (red arrow). Two additional splices sites are conserved in the two *5-ht2α* genes only (green arrows) whereas the two *5-ht2β* genes have one additional splice site in common (blue arrow).
(PDF)

Figure S4 **Tissue-specific expression patterns of** ***Am5-ht2*** **receptor genes in drones determined by quantitative real-time PCR.** Transcript levels were normalized to *Amef-1α*. (**A**) Relative levels of receptor-gene mRNAs in the brain of drones (*Am5-ht2α* and *Am5-ht2αΔIII*: n = 10; *Am5-ht2β*: n = 6; *Am5-ht2βΔII*: n = 5). (**B**) Relative levels of receptor-gene mRNAs in the ventral nerve cord of drones (*Am5-ht2α, Am5-ht2αΔIII*, and *Am5-ht2β*: n = 7; *Am5-ht2βΔII*: n = 6). Groups that differed significantly in relative mRNA levels within a given tissue are indicated with different letters above the box plots (p < 0.05, Bonferroni’s multiple comparison test).
(PDF)

Table S1 **Sequences of primers used for full-length cloning of** ***Am5-ht2*** **cDNAs, construction of expression vectors, and production of monoclonal antibodies against Am5-HT_2α_.**
(DOCX)

## References

[1] Berger M, Gray JA, Roth BL (2009) The expanded biology of serotonin. Annu Rev Med 60: 355–366. 19630576 10.1146/annurev.med.60.042307.110802 PMC5864293

[2] Jones BJ, Blackburn TP (2002) The medical benefit of 5-HT research. Pharmacol Biochem Behav 71: 555–568. 11888547 10.1016/s0091-3057(01)00745-6

[3] Pytliak M, Vargová V, Mechírová V, Felšöci M (2011) Serotonin receptors - from molecular biology to clinical applications. Physiol Res 60: 15–25. 20945968 10.33549/physiolres.931903

[4] Hannon J, Hoyer D (2008) Molecular biology of 5-HT receptors. Behav Brain Res 195: 198–213. 18571247 10.1016/j.bbr.2008.03.020

[5] Nichols DE, Nichols CD (2008) Serotonin receptors. Chem Rev 108: 1614–1641. 18476671 10.1021/cr078224o

[6] Komuniecki RW, Hobson RJ, Rex EB, Hapiak VM, Komuniecki PR (2004) Biogenic amine receptors in parasitic nematodes: what can be learned from *Caenorhabditis elegans*? Mol Biochem Parasitol 137: 1–11. 15279946 10.1016/j.molbiopara.2004.05.010

[7] Clark MC, Dever TE, Dever JJ, Xu P, Rehder V, et al (2004) Arthropod 5-HT_2_ receptors: a neurohormonal receptor in decapod crustaceans that displays agonist independent activity resulting from an evolutionary alteration to the DRY motif. J Neurosci 24: 3421–3435. 15056722 10.1523/JNEUROSCI.0062-04.2004 PMC6730010

[8] Spitzer N, Edwards DH, Baro DJ (2008) Conservation of structure, signaling and pharmacology between two serotonin receptor subtypes from decapod crustaceans, *Panulirus interruptus* and *Procambarus clarkii* . J Exp Biol 211: 92–105. 18083737 10.1242/jeb.012450 PMC4019008

[9] Blenau W, Baumann A (2001) Molecular and pharmacological properties of insect biogenic amine receptors: lessons from *Drosophila melanogaster* and *Apis mellifera* . Arch Insect Biochem Physiol 48: 13–38. 11519073 10.1002/arch.1055

[10] Hauser F, Cazzamali G, Williamson M, Blenau W, Grimmelikhuijzen CJ (2006) A review of neurohormone GPCRs present in the fruitfly *Drosophila melanogaster* and the honey bee *Apis mellifera* . Prog Neurobiol 80: 1–19. 17070981 10.1016/j.pneurobio.2006.07.005

[11] Blenau W, Thamm M (2011) Distribution of serotonin (5-HT) and its receptors in the insect brain with focus on the mushroom bodies. Lessons from *Drosophila melanogaster* and *Apis mellifera* . Arthropod Struct Dev 40: 381–394. 21272662 10.1016/j.asd.2011.01.004

[12] Saudou F, Boschert U, Amlaiky N, Plassat JL, Hen R (1992) A family of *Drosophila* serotonin receptors with distinct intracellular signalling properties and expression patterns. EMBO J 11: 7–17. 1310937 10.1002/j.1460-2075.1992.tb05021.x PMC556419

[13] Colas JF, Launay JM, Kellermann O, Rosay P, Maroteaux L (1995) *Drosophila* 5-HT_2_ serotonin receptor: coexpression with fushi-tarazu during segmentation. Proc Natl Acad Sci U S A 92: 5441–5445. 7777527 10.1073/pnas.92.12.5441 PMC41710

[14] Witz P, Amlaiky N, Plassat JL, Maroteaux L, Borrelli E, et al (1990) Cloning and characterization of a *Drosophila* serotonin receptor that activates adenylate cyclase. *Proc Natl Acad Sci U S A* 87: 8940–8944. 2174167 10.1073/pnas.87.22.8940 PMC55076

[15] Scheiner R, Baumann A, Blenau W (2006) Aminergic control and modulation of honeybee behaviour. Curr Neuropharmacol 4: 259–276. 18654639 10.2174/157015906778520791 PMC2475800

[16] Wright GA, Mustard JA, Simcock NK, Ross-Taylor AA, McNicholas LD, et al (2010) Parallel reinforcement pathways for conditioned food aversions in the honeybee. Curr Biol 20: 2234–2240. 21129969 10.1016/j.cub.2010.11.040 PMC3011020

[17] Wright GA (2011) The role of dopamine and serotonin in conditioned food aversion learning in the honeybee. Commun Integr Biol 4: 18–320. 10.4161/cib.4.3.14840 PMC3187896 21980568

[18] Ellen CW, Mercer AR (2012) Modulatory actions of dopamine and serotonin on insect antennal lobe neurons: insights from studies *in vitro* . J Mol Histol 43: 401–404. 22430182 10.1007/s10735-012-9401-7

[19] Blenau W, Thamm M, Baumann A (2013) Serotonin in insects: distribution, biosynthesis, uptake, inactivation, receptors, functions, and implications for human health. In: Hall FS, New York: NOVA Publishers. pp. 1–26.

[20] Schürmann FW, Klemm N (1984) Serotonin-immunoreactive neurons in the brain of the honeybee. J Comp Neurol 225: 570–580. 6376546 10.1002/cne.902250407

[21] Seidel C, Bicker G (1996) The developmental expression of serotonin-immunoreactivity in the brain of the pupal honeybee. Tissue Cell 28: 663–672. 18621341 10.1016/s0040-8166(96)80070-x

[22] Taylor DJ, Robinson GE, Logan BJ, Laverty R, Mercer AR (1996) Changes in brain amine levels associated with the morphological and behavioural development of the worker honeybee. J Comp Physiol [A] 170: 715–721. 10.1007/BF00198982 1432851

[23] Wagener-Hulme C, Kuehn JC, Schulz DJ, Robinson GE (1999) Biogenic amines and division of labor in honey bee colonies. J Comp Physiol [A] 184: 471–479. 10.1007/s003590050347 10377980

[24] Schulz DJ, Robinson GE (1999) Biogenic amines and division of labor in honey bee colonies: behaviorally related changes in the antennal lobes and age-related changes in the mushroom bodies. J Comp Physiol [A] 184: 481–488. 10.1007/s003590050348 10377981

[25] Blenau W, Erber J, Baumann A (1998) Characterization of a dopamine D1 receptor from *Apis mellifera*: cloning, functional expression, pharmacology, and mRNA localization in the brain. J Neurochem 70: 15–23. 9422342 10.1046/j.1471-4159.1998.70010015.x

[26] Blenau W, Balfanz S, Baumann A: Amtyr1 (2000) characterization of a gene from honeybee (*Apis mellifera*) brain encoding a functional tyramine receptor. J Neurochem 74: 900–908. 10693920 10.1046/j.1471-4159.2000.0740900.x

[27] Grohmann L, Blenau W, Erber J, Ebert PR, Strünker T, et al (2003) Molecular and functional characterization of an octopamine receptor from honeybee (*Apis mellifera*) brain. J Neurochem 86: 725–735. 12859685 10.1046/j.1471-4159.2003.01876.x

[28] Humphries MA, Mustard JA, Hunter SJ, Mercer A, Ward V, et al (2003) Invertebrate D2 type dopamine receptor exhibits age-based plasticity of expression in the mushroom bodies of the honeybee brain. J Neurobiol 55: 315–330. 12717701 10.1002/neu.10209

[29] Mustard JA, Blenau W, Hamilton IS, Ward VK, Ebert PR, et al (2003) Analysis of two D1-like dopamine receptors from the honey bee *Apis mellifera* reveals agonist-independent activity. Brain Res Mol Brain Res 113: 67–77. 12750008 10.1016/s0169-328x(03)00091-3

[30] Beggs KT, Hamilton IS, Kurshan PT, Mustard JA, Mercer AR (2005) Characterization of a D2-like dopamine receptor (AmDOP3) in honey bee, *Apis mellifera* . Insect Biochem Mol Biol 35: 873–882. 15944083 10.1016/j.ibmb.2005.03.005

[31] Beggs KT, Tyndall JD, Mercer AR (2011) Honey bee dopamine and octopamine receptors linked to intracellular calcium signaling have a close phylogenetic and pharmacological relationship. PLoS One 6: e26809. 22096499 10.1371/journal.pone.0026809 PMC3214027

[32] Thamm M, Balfanz S, Scheiner R, Baumann A, Blenau W (2010) Characterization of the 5-HT_1A_ receptor of the honeybee (*Apis mellifera*) and involvement of serotonin in phototactic behavior. Cell Mol Life Sci 67: 2467–2479. 20349263 10.1007/s00018-010-0350-6 PMC11115497

[33] Schlenstedt J, Balfanz S, Baumann A, Blenau W (2006) Am5-HT_7_: molecular and pharmacological characterization of the first serotonin receptor of the honeybee (*Apis mellifera*). J Neurochem 98: 1985–1998. 16945110 10.1111/j.1471-4159.2006.04012.x

[34] Colas JF, Launay JM, Vonesch JL, Hickel P, Maroteaux L (1999) Serotonin synchronises convergent extension of ectoderm with morphogenetic gastrulation movements in *Drosophila* . Mech Dev 87: 77–91. 10495273 10.1016/s0925-4773(99)00141-0

[35] Schaerlinger B, Launay JM, Vonesch JL, Maroteaux L (2007) Gain of affinity point mutation in the serotonin receptor gene 5-HT_2Dro_ accelerates germband extension movements during *Drosophila* gastrulation. Dev Dyn 236: 991–999. 17366631 10.1002/dvdy.21110

[36] Röser C, Jordan N, Balfanz S, Baumann A, Walz B, et al (2012) Molecular and pharmacological characterization of serotonin 5-HT_2α_ and 5-HT_7_ receptors in the salivary glands of the blowfly *Calliphora vicina* . PLoS One 7: e49459. 23145175 10.1371/journal.pone.0049459 PMC3493529

[37] Honeybee Genome Sequencing Consortium (2006) Insights into social insects from the genome of the honeybee *Apis mellifera* . Nature 443: 931–949. 17073008 10.1038/nature05260 PMC2048586

[38] Tamura K, Dudley J, Nei M, Kumar S: MEGA4 (2007) Molecular Evolutionary Genetics Analysis (MEGA) software version 4.0. Mol Biol Evol 24: 1596–1599. 17488738 10.1093/molbev/msm092

[39] Chen C, Okayama H (1987) High-efficiency transformation of mammalian cells by plasmid DNA. Mol Cell Biol 7: 2745–2752. 3670292 10.1128/mcb.7.8.2745-2752.1987 PMC367891

[40] Balfanz S, Ehling P, Wachten S, Jordan N, Erber J, et al (2012) Functional characterization of transmembrane adenylyl cyclases from the honeybee brain. Insect Biochem Mol Biol 42: 435–445. 22426196 10.1016/j.ibmb.2012.02.005

[41] Käll L, Krogh A, Sonnhammer EL (2004) A combined transmembrane topology and signal peptide prediction method. J Mol Biol 338: 1027–1036. 15111065 10.1016/j.jmb.2004.03.016

[42] Moro O, Lameh J, Högger P, Sadée W (1993) Hydrophobic amino acid in the i2 loop plays a key role in receptor-G protein coupling. J Biol Chem 268: 22273–22276. 8226735

[43] Korf I (2004) Gene finding in novel genomes. BMC Bioinformatics 5: 59. 15144565 10.1186/1471-2105-5-59 PMC421630

[44] Wang J, Li S, Zhang Y, Zheng H, Xu Z, et al (2003) Vertebrate gene predictions and the problem of large genes. Nat Rev Genet 4: 741–749. 12951575 10.1038/nrg1160

[45] Huang X, Xiao H, Rex EB, Hobson RJ, Messer WS, et al (2002) Functional characterization of alternatively spliced 5-HT_2_ receptor isoforms from the pharynx and muscle of the parasitic nematode, *Ascaris suum* . J Neurochem 83: 249–258. 12423236 10.1046/j.1471-4159.2002.01067.x

[46] Guest PC, Salim K, Skynner HA, George SE, Bresnick JN, et al (2000) Identification and characterization of a truncated variant of the 5-hydroxytryptamine_2A_ receptor produced by alternative splicing. Brain Res 876: 238–244. 10973616 10.1016/s0006-8993(00)02664-0

[47] Canton H, Emeson RB, Barker EL, Backstrom JR, Lu JT, et al (1996) Identification, molecular cloning, and distribution of a short variant of the 5hydroxytryptamine_2C_ receptor produced by alternative splicing. Mol Pharmacol 50: 799–807. 8863824

[48] Wang Q, O'Brien PJ, Chen CX, Cho DS, Murray JM, et al (2000) Altered G protein-coupling functions of RNA editing isoform and splicing variant serotonin_2C_ receptors. J Neurochem 74: 1290–1300. 10693963 10.1046/j.1471-4159.2000.741290.x

[49] Kilpatrick GJ, Dautzenberg FM, Martin GR, Eglen RM (2000) 7TM receptors: the splicing on the cake. Trends Pharmacol Sci 20: 294–301. 10.1016/s0165-6147(99)01355-3 10390648

[50] Kristiansen K, Kroeze WK, Willins DL, Gelber EI, Savage JE, et al (2000) A highly conserved aspartic acid (Asp-155) anchors the terminal amine moiety of tryptamines and is involved in membrane targeting of the 5-HT_2A_ serotonin receptor but does not participate in activation via a “salt-bridge disruption” mechanism. J Pharmacol Exp Ther 293: 735–746. 10869371

[51] Sugiura M, Fuke S, Suo S, Sasagawa N, Van Tol HH, et al (2005) Characterization of a novel D2-like dopamine receptor with a truncated splice variant and a D1-like dopamine receptor unique to invertebrates from *Caenorhabditis elegans* . J Neurochem 94: 1146–1157. 16001968 10.1111/j.1471-4159.2005.03268.x

[52] Lee SP, O'Dowd BF, Ng GY, Varghese G, Akil H, et al (2000) Inhibition of cell surface expression by mutant receptors demonstrates that D2 dopamine receptors exist as oligomers in the cell. Mol Pharmacol 58: 120–128. 10860933 10.1124/mol.58.1.120

[53] Ohashi K, Sasaki M, Sasagawa H, Nakamura J, Natori S, et al (2000) Functional flexibility of the honey bee hypopharyngeal gland in a dequeened colony. Zoolog Sci 17: 1089–1094. 18522463 10.2108/zsj.17.1089

[54] Deseyn J, Billen J (2005) Age-dependent morphology and ultrastructure of the hypopharyngeal gland of *Apis mellifera* workers (Hymenoptera, Apidae). Apidologie 36: 49–57.

[55] Drapeau MD, Albert S, Kucharski R, Prusko C, Maleszka R (2006) Evolution of the Yellow/Major Royal Jelly Protein family and the emergence of social behavior in honey bees. Genome Res 16: 1385–1394. 17065613 10.1101/gr.5012006 PMC1626640

[56] Maleszka R (2008) Epigenetic integration of environmental and genomic signals in honey bees: the critical interplay of nutritional, brain and reproductive networks. Epigenetics 3: 188–192. 18719401 10.4161/epi.3.4.6697

[57] Goldman TD, Arbeitman MN (2007) Genomic and functional studies of *Drosophila* sex hierarchy regulated gene expression in adult head and nervous system tissues. PLoS Genet 3: e216. 18039034 10.1371/journal.pgen.0030216 PMC2082469

