# Supplementary material for: Function and Distribution of 5-HT2 Receptors in the Honeybee (Apis mellifera)
Source: PLoS One. 2013 Dec 6;8(12):e82407. doi: 10.1371/journal.pone.0082407 (PMC3855752; doi:10.1371/journal.pone.0082407)
Supplement: Figure S1 — Sequences of primers and TaqMan probes (including 5’- and 3’-modifications; see material and methods) used for qPCR assays and the expected length of the resulting amplicons. The positions of primers on the cDNAs are schematically shown by open arrows, TaqMan probes are indicated as asterisks. (PDF) [file pone.0082407.s001.pdf]

# Figure S1

| transcript | primers and probes (5‘ → 3‘) | amplicon size |
|---|---|---|
| *Am5-ht2α* | sense: GTCTCCAGCTCGATCACGGTT<br>antisense: GGGTATGTAGAAGGCGATCAGAGA<br>probe: Cy5-CGTGATCAACAACAGAGCGTTTTTCGT-BBQ | 126 bp |

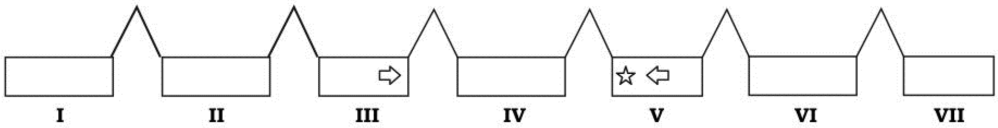


| transcript | primers and probes (5‘ → 3‘) | amplicon size |
|---|---|---|
| *Am5-ht2αΔIII* | sense: CCAGGCTTCTTGGGCATAAT<br>antisense: GGGTATGTAGAAGGCGATCAGAGA<br>probe: Cy5-CGTGATCAACAACAGAGCGTTTTTCGT-BBQ | 114 bp |

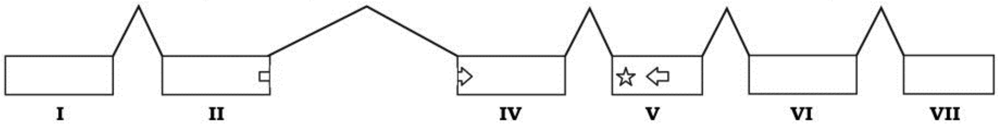


| transcript | primers and probes (5‘ → 3‘) | amplicon size |
|---|---|---|
| *Am5-HT2β* | sense: GAGTTTGCCACTCAGTCTGATGTACT<br>antisense: GCAGATTATGCTGCCGATCAAC<br>probe: Cy5.5-TGGTGGACGGTGCCTGTCAAA-BBQ | 109 bp |

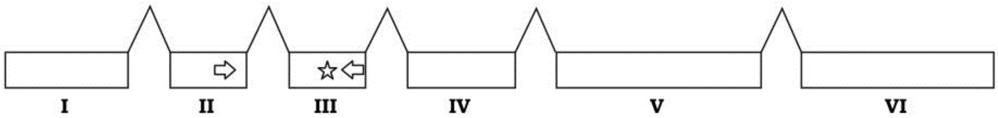


| transcript | primers and probes (5‘ → 3‘) | amplicon size |
|---|---|---|
| *Am5-HT2βΔII* | sense: CACTTTGGTCAGAGGAGGACGA<br>antisense: GCAGATTATGCTGCCGATCAAC<br>probe: Cy5.5-TGGTGGACGGTGCCTGTCAAA-BBQ | 92 bp |

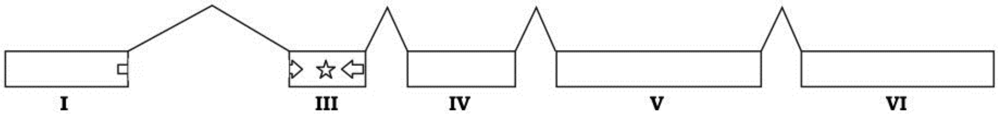


| transcript | primers and probes (5‘ → 3‘) | amplicon size |
|---|---|---|
| *Amef-1α* | sense: GAACATTTCTGTGAAAGAGTTGAGGC<br>antisense: TTTAAAGGTGACACTCTTAATGACGC<br>probe: 6FAM-ACCGAGGAGAATCCGAAGAGCATCAA-BBQ | 394 bp |
