# Supplementary figures and images for: Function and Distribution of 5-HT2 Receptors in the Honeybee (Apis mellifera)
Source: PLoS One. 2013 Dec 6;8(12):e82407. doi: 10.1371/journal.pone.0082407 (PMC3855752; doi:10.1371/journal.pone.0082407)

Figure S2

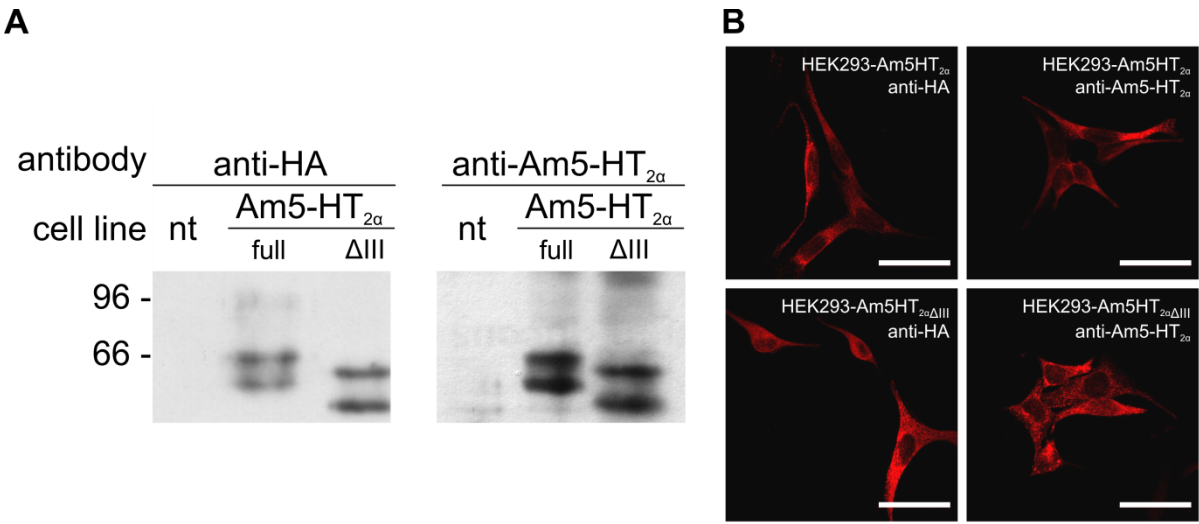

Supplement: Figure S2 — Western blot and immunocytochemical analyses of Am5-HT2α- and Am5-HT2αΔIII-expressing cell lines. Anti-Am5-HT2α antibodies were raised against a fusion protein containing part of the third cytoplasmic loop (CPL3; amino acid Arg340 to Glu452; see Fig. 1). The cDNA fragment was amplified by PCR with specific primers (Table S1). The fragment was cloned into pMAL-c2X vector (New England Biolabs, Frankfurt, Germany). The fusion protein was over-expressed in E. coli BL21 CP and purified by amylose affinity-chromatography (New England Biolabs). In collaboration with the Nachwuchsgruppe Antikörper-Technologien (University of Potsdam, Germany), the fusion protein was used to immunize mice and to raise monoclonal antibodies. A second fusion protein containing a His-tag attached to the same receptor fragment was expressed from pET-30a vector (Novagen, Darmstadt, Germany) and used for testing the specificity of the monoclonal antibodies. Membrane proteins (10 µg protein per lane) of human embryonic kidney cells (HEK 293) expressing Am5-HT2α-HA and Am5-HT2αΔIII-HA receptors (see below) were isolated as previously described (Thamm et al., 2010). Proteins were separated by SDS polyacrylamide gel electrophoresis on 10% or 12% gels and transferred to polyvinylidene fluoride membranes (Roth, Karlsruhe, Germany). These membranes were blocked with 5% (w/v) dry milk in Tris-buffered saline containing Tween 20 (TBS-T, 10 mM Tris-HCl, pH 7.5, 150 mM NaCl, 0.01% Tween 20) for 30 min at room temperature, incubated either with specific anti-HA antibodies (Anti-HA High Affinity, Roche, Penzberg, Germany; dilution 1∶5,000) or with receptor-specific antibodies (dilution 1∶100) in TBS-T, washed with TBS-T, and finally incubated with secondary antibodies (1∶5,000, anti-rat-HRP; American Qualex, La Mirada, USA; 1∶200, anti-mouse Alexa568; Invitrogen) for 1 h. Signals were visualized by enhanced chemiluminescence. (A) Western blot analyses of membrane proteins (10 µg per lane) of non-transfected HE [file pone.0082407.s002.pdf]

Figure S4

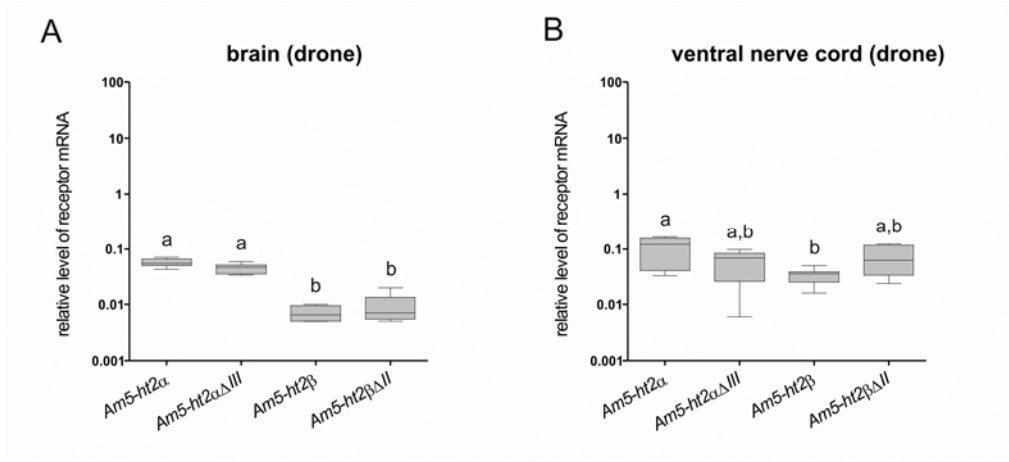

Supplement: Figure S4 — Tissue-specific expression patterns of Am5-ht2 receptor genes in drones determined by quantitative real-time PCR. Transcript levels were normalized to Amef-1α. (A) Relative levels of receptor-gene mRNAs in the brain of drones (Am5-ht2α and Am5-ht2αΔIII: n = 10; Am5-ht2β: n = 6; Am5-ht2βΔII: n = 5). (B) Relative levels of receptor-gene mRNAs in the ventral nerve cord of drones (Am5-ht2α, Am5-ht2αΔIII, and Am5-ht2β: n = 7; Am5-ht2βΔII: n = 6). Groups that differed significantly in relative mRNA levels within a given tissue are indicated with different letters above the box plots (p < 0.05, Bonferroni’s multiple comparison test). (PDF) [file pone.0082407.s004.pdf]
