# Supplementary material for: Function and Distribution of 5-HT2 Receptors in the Honeybee (Apis mellifera)
Source: PLoS One. 2013 Dec 6;8(12):e82407. doi: 10.1371/journal.pone.0082407 (PMC3855752; doi:10.1371/journal.pone.0082407)
Supplement: Figure S3 — Comparison of splice sites in 5-ht2 genes (coding regions) of Apis mellifera and Drosophila melanogaster . Splice donor and acceptor sites were identified using the Splign alignment tool (http://www.ncbi.nlm.nih.gov/sutils/splign/splign.cgi). In the mRNA sequence alignments (A+B), which were created using the program BioEdit version 7.1.3.0, splice sites are indicated by +++ and highlighted in yellow and green for 5-ht2 genes of the honeybee and D. melanogaster, respectively. (A) Am5-ht2α is the orthologue of the Dm5-ht2α gene (CG1056) with which it has three introns in common. (B) Am5-ht2β is the orthologue of the D. melanogaster CG42796 gene with which it has two introns in common. (C) A pictogram of a GPCR with its seven transmembrane segments (green bars) is displayed. Arrows point to the relative positions of splice sites in the primary structures of the receptors. For each exon, the last amino acid residue is indicated and numbered according to its position in the deduced amino acid sequence. One splice site is conserved in all four 5-ht2 genes (red arrow). Two additional splices sites are conserved in the two 5-ht2α genes only (green arrows) whereas the two 5-ht2β genes have one additional splice site in common (blue arrow). (PDF) [file pone.0082407.s003.pdf]

Figure S3

A

```
Am5-ht2α  ATGGAGGGG------------------------------------------------------------- 9
Dm5-ht2α  ATGGAGATGCAAAGCTACTCTGGTCGTGGACCATCGACAGGCACATCAGAAACAGCAACAGCAACAGCAG 70

Am5-ht2α  ---------------------------------------------------------------------- 9
Dm5-ht2α  CAGTTGCATCTGCATCTGCATCAGACACAGCAGCAACATTCACACTGCGACACTTCAATTGTCGGCTGGA 140

Am5-ht2α  -------------------------------------GATCTGATCGCCTCTTGGACCGGCAACCTGACC 42
Dm5-ht2α  AGAGGCGGATTGGCCTGGGCCTCGCCAGCCGTTTATTGACTGCGAAGGCAGCCACACCAACAGCACATCC 210

Am5-ht2α  GCGATGTTGCACCACCTGCCCTGCAACGACACCTTCCTCCACGGA------------------------- 87
Dm5-ht2α  TACACCCCCAGCAGCCTGCCCACCAGCAAAAACCTCATCGAGGGACCCAACAACACATTCCAGTGTGCAC 280

Am5-ht2α  -----------------------------------------TGCGCGGAGGAGTGTGGACTCGCCTCG-- 114
Dm5-ht2α  GGCAAGCTATCGACGAAGCGGAGCACAACGCATTCTATAGTTGCGATGGGGAAAGCCCATTGTCTGAAAA 350

Am5-ht2α  -------------------------------------------TCGGGATTCGATCACATGTGCACGGCC 141
Dm5-ht2α  CTGCACTTTGAGGCTGAAGGCGCGACTGTTGCGATGGCCGTACGAGGCGTATGAGCACATTCGACTGGCA 420

Am5-ht2α  GAGAAGGGAAGCTTCGCCTCGTCCATGACTCTGCTCTGCACGCCCTGCGACTACTCCAACTGCGACGTGA 211
Dm5-ht2α  ATCAACGGGGCACTCAGTGATTATGCTAGTGATAAACTTAGT------GATAGTTCGAGCTGCGATGAAG 484

Am5-ht2α  ACGTGTCCTTGGTCCTCAGCGGGCTGGAGAGCGTGGAGGGCGTTTTCCTGTCCAGGTTGCCGAGGTTCGA 281
Dm5-ht2α  GAGGCATGGTGTTGCAATGTAAGCTGGGTCAGGCCCATGAACTATTGCTTTGTCATTTGCAACAACTCGA 554

Am5-ht2α  GTGCAAC------------------------GAGACCGATTTTCCCGATCAG+++--------------- 309
Dm5-ht2α  GAGGGAGCCACTGCGGGAGGTGGGAAGCGCGGAGGAGAGCTTCTCCAACGACAGCCTTCTGCAGGCCTTT 624

Am5-ht2α  ---------------------------------------------------------------------- 309
Dm5-ht2α  CAAATCGAACTGGGCGCAGAGCGCGATGGACTCTGGTCCTTCCTTAATCTAAGCGAGCTAATGGAGCCCG 694

Am5-ht2α  -----------------ATATTGGAGTGCACGGCGACT---------------ACGGAGAACGCGACTAC 347
Dm5-ht2α  ATCACGGATTCCTGGGCATCATGAATGTCACCAACACTAGCTTAGACATGTTTTTGCCGAACCTTACAGC 764

Am5-ht2α  GGAGGATCTGACTATCTCTTGCTCGCACAGGGCGAAGGGCTTGAATCCGGCTGGGAGCAGA--------- 408
Dm5-ht2α  CCTGAACACGACCTCCACGGCCTCCACTGCAGACATGGCCACCGAGTTCGCGGTAACAAAGAGCTTTCTG 834

Am5-ht2α  ---------GGGCGACGTCTCGACTGGAACTTTCTCTTCGTTGCTGTATTTATCGTGGCGGGTGGCCTGG 469
Dm5-ht2α  GACTACAGTCCCCATGGCTACGATTTCCTATTCCTGTTCGTGGTGTTCTTTATCTTCGCGGGTGGCCTGG 904

Am5-ht2α  GGAACATACTCGTCTGTCTCGCTGTGGGGCTCGATAAAAGGCTGCACAACGTCACCAATTACTTCCTTTT 539
Dm5-ht2α  GTAATATCCTTGTGTGCCTAGCCGTTGCCTTGGACCGGAAACTGCAGAATGTGACTAACTACTTCCTGTT 974

Am5-ht2α  GTCTCTCGCGGTGGCCGATTTACTCGTCAGCCTGTTCGTCATGCCTCTGGGCGCGATCCCAGGCTTCTTG 609
Dm5-ht2α  TTCCCTGGCTATAGCCGATCTCCTGGTCAGTTTGTTTGTGATGCCCATGGGCGCGATACCAGCTTTTTTG 1044

Am5-ht2α  G+++GATACTGGCCATTCGGAGTGGTGTGGTGTAACGTGTACGTGACGTGCGATGTGCTGGCTTGTTCCG 676
Dm5-ht2α  G+++GGTATTGGCCACTTGGCTTTACCTGGTGCAACATTTATGTGACGTGTGACGTACTGGCCTGCTCAT 1111

Am5-ht2α  CGAGCATAATGCACATGTGCTTCATCTCGTTGGGCCGGTATCTCGGCATCCGGAATCCTTTGCGGACGAG 746
Dm5-ht2α  CCAGTATATTGCACATGTGCTTCATCGGTTTGGGACGCTATATGGGAATAAGAAATCCATTGGGCTCGAG 1181

Am5-ht2α  GCACACGTCCACCAAACGGATGGTCGGCTTCAAGATAGCGGCTGTGTGGTTGCTCGCCATGTTGGTCTCC 816
Dm5-ht2α  GCATCGATCTACGAAACGATTGACTGGTATAAAGATAGCCATTGTTTGGGTAATGGCCATGATGGTATCC 1251

Am5-ht2α  AGCTCGATCACGGTTTTGG+++GCATAATAAATCCTCTAAACATCATGCCACGGCCGGGAGCGTGCGTGA 883
Dm5-ht2α  AGTTCAATAACCGTTCTTG+++GTCTGGTCAATGAGAAGAGCATAATGCCCGAGCCTAACATATGCGTTA 1318

Am5-ht2α  TCAACAACAGAGCGTTTTTCGTGTTCGGCTCTCTGATCGCCTTCTACATACCCATGATCGTCATGGTAGC 953
Dm5-ht2α  TCAACAATCGCGCCTTTTTCGTGTTTGGATCTCTGGTAGCTTTTTATATACCCATGCTGATGATGGTTAC 1388

Am5-ht2α  CACTTACATTCTGACGGTGCAGCTGCTCAGGCAAAAAGCCCGTTTCATCGCGGAACATCCTGAACGAGAT 1023
Dm5-ht2α  CACATACGCATTGACGATTCCCCTCCTCCGGAAGAAAGCGCGATTTGCCGCCGAGCACCCGGAAAGTGAA 1458
```

```
Am5-ht2α   CAATTCCGAAG---GCTGGGAGGC---------------------------------------------- 1044
Dm5-ht2α   CCTTTTCGCAG+++GTTGGGTGGACGCTTCACCCTAAGGCCGCAGCACAGCCAGCAGCAGTTGCAGATGT 1525

Am5-ht2α   -----------------------------------------------------AGATATTTCTCGACGAG 1061
Dm5-ht2α   TCAGTAGCTTTTCTGGCAGCAATAACAAATTCCTATCAATGGGCGACGGCAATCGCAACTTCAACACTGA 1595

Am5-ht2α   G------------------GCTTCCTCCACCGCCACCTCGACCGTGGAAACCGCCACCCCT--------- 1104
Dm5-ht2α   AGGAGAGATGGAGGAGGGTGGAGCTCCTAGCAGAAGGAGTGGCGTAGAGCCCGCCGAACGACCTTTGATG 1665

Am5-ht2α   ------------TCGTCGTCGCGG------------TATACGTGGAGG---------------ACTTCCG 1135
Dm5-ht2α   CAACAGCGAACGGCGAGCAGCAGGAGCATGGGCACGGTTAGTTTCCGTAATGTCGTGAACGGGACTAGCG 1735

Am5-ht2α   GCGGTGTCGGAAGCGACAG+++TTTAACCAAATTAAACGCGGAGGCTCTGTGCCGT-------------- 1188
Dm5-ht2α   GAGCCGCGGGAAGTGGCG---CAGGACGACAGGCACTGCCCACAGCAGCTTCCGGTTCTCTGGCGCCGG 1802

Am5-ht2α   ---------------------------------------------------------------------- 1188
Dm5-ht2α   CATCCTTCGGCACTCGTCGTCGTCTCCCGCCTCGAGCTGCCACTCCACCAGCACGTCGCGTTCGAGCTCC 1872

Am5-ht2α   ------CGCAAACATAAC---------------------------------------------------- 1200
Dm5-ht2α   TTCTGGCGCAAACACGGCGGCTATCCAAACCTAATGGACAG+++ACTGGCCATACATTTAAGACTTTCTT 1939

Am5-ht2α   -----------------------AGGTGCAGGGGGGCAAGGTCGGCCGCAAAGTCTCAACCGCAGTTGAA 1247
Dm5-ht2α   TGGTACCACCCAGTAGCCATCCAAATCGACGGGCATCTGTGCGTGTCACCATCTCCCAGCCACAATTGGG 2009

Am5-ht2α   TTTCGCCGTGAACGGG------------------------------------------------------ 1263
Dm5-ht2α   TTACCCGACCAATGTAGGTGGAAATGGAGGTAGAACTGTTGGAGCGACGGAGGTGGGGGCTGGTGGCAAT 2079

Am5-ht2α   ------------------GCGAGTAACCCGGCCGGAAGCGGAACCGGGGCGACGAGACTGGCGACGAAAG 1315
Dm5-ht2α   GGCTCATCAGCCGGCAGAACGAGCAACTCATCTCTTGGAGGCACGAGCTTGATGAAAATCGCAACCATCC 2149

Am5-ht2α   TGGATCAGGCGACGCAAACGCCGGAGAATATCGGC----------------------------------- 1350
Dm5-ht2α   AGGGACCGATACTGAACCAAAGTCAGGCAGCTGGAGGCAATCACATGGTGCAAAAGGTGAAGCCGACGGC 2219

Am5-ht2α   ----------CGTGAAACAAGGAATTTCACTCTGAAGGCTTTGAAGCTCCAATTAAAC---GTCACTCCC 1407
Dm5-ht2α   TTTAAAACCGGAAGAACCACAGCGCAACAAAGTGCGGCCCTTTAAATTTGCCTTCAACCGCGTTGCCACG 2289

Am5-ht2α   AACACCCTGAATCTCAG+++GTTTCTCCCCGGTAGAACGAAGAGGAGATCGTTGGCCGCGAACGCCGTTG 1474
Dm5-ht2α   CCCACCCTCAATTTGCG+++TTTTCTAAACAATCGCAGTAAACGAAACAGCTTGTCGGCCAATGCCGTTG 2356

Am5-ht2α   CAACGGAGCAGAAAGCCAGCAAGGTGTTGGGCCTGGTTTCTTCATGTTCGTGCTCTGCTGGGCCCCGTT 1544
Dm5-ht2α   CTACGGAGCAGAAGGCCACCAAGGTGCTCGGATTGGTGTTTTTCACCTTCGTTCTGTGCTGGTCGCCTTT 2426

Am5-ht2α   CTTTCTACTAAACATATTTTTCGCCGCGTGCCCCGAATGCTCGGTCCCGGTACACGTGGTCAACGTGTGT 1614
Dm5-ht2α   CTTCATCTTGAACATTATATTTGCCGCATGCCCCGAGTGCCAAGTGCCGGAGCACGTGGTGAATACCTGC 2496

Am5-ht2α   CTGTGGCTCGGATACGTGTCCTCCACGATCAATCCGGTCATCTACACCATCTTCAATAGAACGTTCAGGG 1684
Dm5-ht2α   CTTTGGCTTGGCTATGTCTCCTCGACGATCAATCCCATCATCTACACCATATTCAATCGCACGTTCCGGG 2566

Am5-ht2α   CTGCGTTCATCAGATTGTTGAAGTGCAAGTGTTCCAG+++GTCAGCAAGGCCGAGGCATCGTTCAGTGAC 1751
Dm5-ht2α   CGGCCTTTATCCGCCTGCTCAAGTGCAACTGCAAACG---GTGA                           2607

Am5-ht2α   GGAGAGTGGACGAAACGCGATGAGCCTGTGCACCCCGACCGCGCTGCCATTGGTCATTAGTCTTCAGGGT 1821
Am5-ht2α   ACGCCGTTGTTGACGCCAACACCGAATCCGACGGCGGCCACGCCGGCCTCGGCAACCTCCTACGTGACGA 1891
Am5-ht2α   TGATGCATCGCACGCCAACCTCCCTTTACCGTGACACTTTTCATCAACACGAGCACGATCAGAATTGTTG 1961
Am5-ht2α   A 1962
```

B

```
Am5-ht2β   ATGATCGACTCGTCGACGACGACGATCGATCGCGCGGATCCTCACGAGGCCGTGGTCGACAGCTGTTACC 70
CG42796    ATGGAAGAGGATGTGTATGCCTCGCTAGGTGCCTACAACGACAGCGGTGGCGACGATTGGAGCAGCTCGG 70

Am5-ht2β   AGGATCTTTTGCTGTTGGCGAACGAC------AGCAGCGCGCTTTGCAACGGATCAGGAGGATTGGACGT 134
CG42796    AGCACCTGGTCCTGTGGGAGGAGGATGAGACGCAGCGAACGACTGCTAATGCCACCAGTCGGCATAATCA 140

Am5-ht2β   ACTGAACGACACC------GTCGATCTAGAGATTGACCCGGTGAACGGGACACGGTTGGACGTTGACGGA 198
CG42796    ACTGCATGTGGCCAGGTGGAATGCCACCGGCAATGCGACCATCAGCGCGACCTTCGAGGACGTACCCTTC 210

Am5-ht2β   ---TTGAACAATTGGTGGGCGATGCTCGCCTTGGTCCTGGTGTTGGGCACAGCAGCCGGCAATATTTTGG 265
CG42796    GATGCGAACAACTACTGGGCCCTATTGGCCCTCGTCCTCGTCCTGGGAACAGCGGCCGGGAACATCCTTG 280

Am5-ht2β   TGTGCTTGGCCATTGCCAGGGAGAGGAGATTGCAGAACGTCACCAATTATTTCCTGATGAGCTTGGCGAT 335
CG42796    TCTGCCTGGCCATCGCCTGGGAGCGCCGGCTGCAGAATGTGACCAACTACTTCCTTATGTCGCTGGCCAT 350

Am5-ht2β   CACGGATCTGCTCGTAGCCGTGCTTGTAATGCCTCTTGGAATACTCACTTTGGTCAGAG+++GATATTTC 402
CG42796    CACCGATCTCATGGTCGCCGTGCTGGTCATGCCGCTGGGCATCCTTACGCTGGTGAAAG+++GCTACTTT 417

Am5-ht2β   CCTCTGCCGTCAGTTTACTGCCTCGCTTGGATCTGCCTGGATGTATTATTATGCACAGCGAGCATCATGC 472
CG42796    CCTTTGGGCTCGGAGCACTGCCTCACCTGGATCTGCCTGGATGTACTCTTCTGCACGGCCAGCATCATGC 487

Am5-ht2β   ACTTGTGCACCATAAGCGTGGACCGATACCTCAGTCTACGGTATCCCATGAAATTCGGTCGGAATAAGAC 542
CG42796    ACCTGTGCACCATCTCCGTGGACAGATATTTATCGCTACGATACCCAATGCGATTTGGCCGCAATAAAAC 557

Am5-ht2β   GAGGCGGCGGGTCATGTTGAAAATCAGCTTCGTCTGGGTTCTCAGCATCGCGATGAGTTTGCCACTCAGT 612
CG42796    ACGTCGGCGGGTCACTCTCAAAATTGTTTTCGTTTGGCTTCTGAGCATCGCCATGAGCTTGCCCTTGAGC 627

Am5-ht2β   CTGATGTACTCGAAG+++GAGGACGACTCGGTGCTGGTGGACGGTGCCTGTCAAATACCGGATCCCCTGT 679
CG42796    TTGATGTACTCCAAG+++AATCACGCCTCGGTGCTGGTGAATGGAACTTGCCAGATACCGGATCCGGTGT 694

Am5-ht2β   ACAAGTTGATCGGCAGCATAATCTGCTTTTACATACCGTTGGGCGTGATGCTGCTTACGTACGCGTTGAC 749
CG42796    ACAAGCTGGTCGGCTCCATTGTATGCTTCTACATTCCACTGGGCGTGATGCTGCTGACATATTGCCTGAC 764

Am5-ht2β   AGTGAGATTGCTGGCGAAACAGCAACAGAATATAGGAGGA---------------------ACTACTGGT 798
CG42796    CGTCCGACTTTTGGCCCGGCAGCGCCAGAATCTGGGTGGTGGACAGCAGACGGCGGCGGCCACTCCCGGA 834

Am5-ht2β   TGGTCCTCCGGATGGCTGGGTGGCCCGCAGGGCCCATCTTCGGGAG+++GGCTGGACAGGAAAGGCACGT 865
CG42796    TGGGCCAGTGGATGGCTCGGC------CAGGCACCG---------GCCTTGG+++AACGACGATGCACCT 886

Am5-ht2β   GGAAGCGATTTCTGTTGAGCAAAAGCCCGGCCGGAAGCGGCGGTACACCGCAGCACACTTCCGGCACATC 935
CG42796    ---GGCGGCGCCTTTTGAAACCGGGTCCAGGTAACGCCTCCTCGGTGCTGCACGCACACTCGGCCAATTC 953

Am5-ht2β   GACAGACACCGAGCTGACGACACTGGACACTCACGAACTCTGGCTTCCGGAAAGC------------GAG 993
CG42796    AACGGATACGGATCTCAGCACTTTGGACAACCACGAGCTCTGGCTGCCCGATTCCAG+++CATAAAGGAG 1020

Am5-ht2β   CCGCCGCCCTCGGCCATGTTCGCCCTTCACGCTTTCGGCGCAGAGATGCTCAAGTTGTCGAGGGGCCTGG 1063
CG42796    CCAACGCCCACGACCATGACGGCCTTGCATCAGTTCGGTGCCGAGATGCTGAAACTATCCCGAGGCCTGG 1090

Am5-ht2β   AGGGTATCACCAGCCCT---------GGAAGCCCTATGGGC----------------------------- 1095
CG42796    AGTCGGTGGCCTCGTCATCCACCACCGGCTCACCCACCAAATCCGAATTTTCCCTATCGAACCACCTGCA 1160

Am5-ht2β   ----------------ACACCGAGATCCACGCCTCAACACTCGATCCAGCAACATCAGCATCAGCAACAG 1149
CG42796    GCTGCAGTACCCCAGCAGCCCACAGCGATATGCCGCCACCCACCAGCAGTCCCACCACCACAACCACCAG 1230

Am5-ht2β   TCGCACAGGTGCAGCTTCCGTCACGG+++GAGCGGGGAGAGCGGCGGG---------------------- 1194
CG42796    CAGCATCCGGCCGGAGTTTACCACCA---GCAGGCATCGCCCAAAGGTCGCCACGGAACCACAGTTCTGG 1297

Am5-ht2β   -------------------------------------------------------AGCAGCGCTTCCGG 1208
CG42796    GTCTGTCCACCACCACGCTGGGCATCGAGCGGGAGAGCACCAGGAATTCCCTGGCCAGCAGTCGCATGGG 1367

Am5-ht2β   TTCCAGGACGAGCCTCTC---GATCCAAGAAGATCTCAGCTCGCCAACGCCGTGGAAGCATCCACGTCGA 1275
CG42796    CGGCGAACAGAGCGATGG+++GACTCTTTCACAGCTTTCCCAACGATTGCGAGCGTATAAAAGCGACGA 1434

Am5-ht2β   CGCGCGTCCACGTTCAAC-----------------------------GAGGCTCACCTTGAGCGGACCG 1315
CG42796    AGAGCTTCGTCAGCTGTTCCTGGACGTGAGAAACGTGCCGGGCATGAGGACGCAGACGAGGATGTGGAAA 1504
```

```
Am5-ht2β  AGTCGGGATCGCCGAAGACGCCTAGGAAGCGATCGTTCAGCTTCCACGAGCAGCCCGTGTTCGGGCGTGG 1385
CG42796   CGCCCACGTCAACGCTGCGCCGCCACAAGCGCCATAACAGTCTGCCCAAGAACGCACTCTATCCGCGACA 1574

Am5-ht2β  CTCCTCCGTTTCGAGGAAGAGCTCCTCGAAC------------GAGGAAACGCCGCAGAGCCGGAAGTCG 1443
CG42796   CACCACTGTACAGGAGAGCCTCGATGACGACGACGACGAGGAGGAGGAGAGCGCGCAACAGAAGGATCAG 1644

Am5-ht2β  AGCGACAAA------------------------------------------------------------- 1452
CG42796   CAGACGAAGTCGAAGATCTGCCACTCGGACACTGAGCTAGATCCTCCGCAGAAGGCGAAGCAGTGCCACA 1714

Am5-ht2β  -----------------CAGGAGGGTGAGATCGCCCTCCCGCCGCCGTGCACGTGCCCATACTTCGGCGA 1505
CG42796   TAGCCGGGCAGAATCATCAGTCGGACTACTTGCAGCTGCCCTCGGTTTGCACTTGTCCGTATTTTGGGGA 1784

Am5-ht2β  G------------------------------------------------------------------TCG 1509
CG42796   CAGACCGCTCCAGAATTGTGTGAAATCGGCGGAGGTGAAGATTATTTCGTCCGCCTTCCGAGTGACCACC 1854

Am5-ht2β  TCGTCGAGGAGACCCCCGCCCCAACCTTCT---ACCGAGATCGTGATCGTTTCCAGC------------- 1563
CG42796   ACCACGACGGCGGTGAGCAGTTCGCCCAGCGAGATGGAGCTACTCATGTGCAGTAGTGGCAATAAAAAGT 1924

Am5-ht2β  --------------------------------------------------------------------GA 1565
CG42796   CATTGACCTCCAGTGTGAGTGCCGGCATAACAGGTGGACCGGCAGGAGCGGCTCCAGCCACAGTTGGCTC 1994

Am5-ht2β  CACGATGAGGCCGATCTCGGGCAAGAACCTCGAGGCGGCTTTCCTGGGAAGGAGCAAC---AGCGGCAGG 1632
CG42796   CACCCTCAGTCCACACTCTGCTCACAATCAGGGCAGCTCGCTGACAGTTCAGAGCGATGGTAGTGGGTAC 2064

Am5-ht2β  ATGGAGGGCAGCAGAAGC------------------------------TACGAGCTGAATTCCGTTGTCA 1672
CG42796   TTGGCTGCTCCGGGAACTCCTTGTCCTGGCCGACGAAAGTTGAGCATCTCAAAGACCGCCTCGGTGGTCA 2134

Am5-ht2β  CGTGGAGAGGG---------TGCAGGAGAGGGTCTAGTTTGGGAG+++GAAGC----------------- 1713
CG42796   CGTGGGATTCAAGTCGTCATCGTCGGCGGGGCAGCAGTTTTGGCG---GAGTGCGAACATCCCTGCTGTT 2201

Am5-ht2β  -------ACGAGGACCAGCCTGCTGACGTCGAGGCCAGCCCCGATCCGCCGCGCGGCGACGATGCGTGCC 1776
CG42796   GACGCCCACCAAAACAGCGACCACCTCCAACTCATCCACACCGTTGAGAAGATCGGCCACCTTGAGGAGT 2271

Am5-ht2β  CACAAC---------------GGGGCCGCGAGCCTCGGCTCGAGGCAAAGCAGCAATTCGTCGTCCCCGT 1831
CG42796   CACCAGAATATGAACTACCAGGGAGCGGGCGAATGTGGTGGCAAGACCAGGACGACAACATCCTCACCCT 2341

Am5-ht2β  GCCCCCACAGATACCAAACGACAGTACGCAGCCACCACTCGAGGACGAGCAGCGTGGTGTCCCGGAACAG 1901
CG42796   GCATGTTGCAGCGTCAGCAGACGGTGCGATCTCATCACTCGCGGAACTCCAGCGTGATCTCCAGAAACTC 2411

Am5-ht2β  CTCGCGCCACGGTCGGATCATCAGGCTCGAGCAGAAGGCCACCAAGGTGCTCGGGGTGGTGTTCTTCACG 1971
CG42796   CTCGCGCCACGGCAGGATCATCAGGCTGGAGCAGAAGGCCACCAAGGTGCTGGGAGTGGTGTTCTTTACG 2481

Am5-ht2β  TTCGTCATACTGTGGGCGCCGTTCTTCGTTCTCAACCTGATCCCAGCCGTGTGCCCCAACTGCGAGAGAC 2041
CG42796   TTCGTAATCCTCTGGTCGCCATTCTTCGTCCTCAATCTCCTGCCCACGGTGTGCGCGGAGTGCGAGGAAC 2551

Am5-ht2β  AAATCGACCACAAGATATTCGACCTGGCCACCTGGCTGGGCTACGCCAGCTCCATGGTGAACCCCATCTT 2111
CG42796   GAATTAGCCACTGGGTCTTCGACGTGGTCACCTGGCTGGGCTACGCTAGCTCCATGGTGAACCCCATCTT 2621

Am5-ht2β  CTACACCATCTTCAACAAGGTGTTCAGACAAGCGTTCAAGAAGGTGTTGCTCTGCAGGTACAGGAAT--- 2178
CG42796   CTACACCATCTTCAACAAGGTGTTCCGGCAGGCGTTCAAGAAGGTCCTGCTGTGCCGCTATTCGAGCACG 2691

Am5-ht2β  CAAACGTGGAGACCGTCCAGGTAG 2202
CG42796   AGTGCCTGGCGACCGAGCAGATAA 2715
```

C

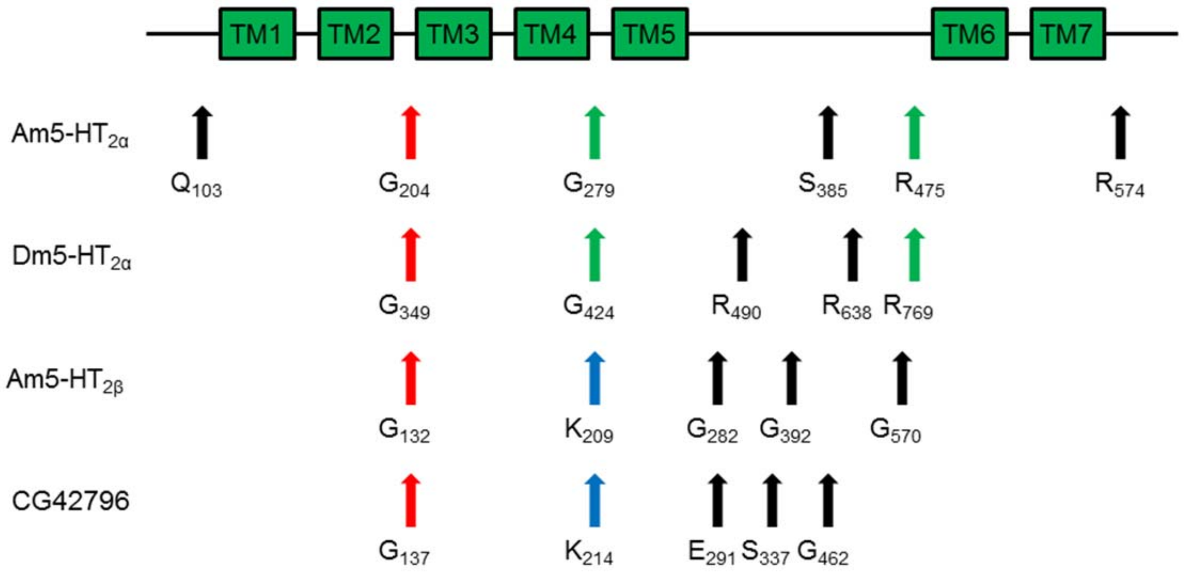
TM1
TM2
TM3
TM4
TM5
TM6
TM7
Am5-HT$_{2\alpha}$
Q_{103} G_{204} G_{279} S_{385} R_{475} R_{574}
Dm5-HT$_{2\alpha}$
G_{349} G_{424} R_{490} R_{638} R_{769}
Am5-HT$_{2\beta}$
G_{132} K_{209} G_{282} G_{392} G_{570}
CG42796
G_{137} K_{214} E_{291} S_{337} G_{462}
