# Supplementary material for: Function and Distribution of 5-HT2 Receptors in the Honeybee (Apis mellifera)
Source: PLoS One. 2013 Dec 6;8(12):e82407. doi: 10.1371/journal.pone.0082407 (PMC3855752; doi:10.1371/journal.pone.0082407)
Supplement: Table S1 — Sequences of primers used for full-length cloning of Am5-ht2 cDNAs, construction of expression vectors, and production of monoclonal antibodies against Am5-HT2α. (DOCX) [file pone.0082407.s005.docx]

**Table S1**

| **purpose** | **receptor** | **primers (5’ 🡪 3’)** |
| --- | --- | --- |
| full-length cloning | Am5-HT_2α_ | sense: TTTGAATTCATGGAGGGGGATCTGATC |
|  |  | antisense: TCAACAATTCTGATCGTGC |
|  | Am5-HT_2β_ | sense: CGTATTCACGCAGATGCC |
|  |  | antisense: CCCTGGTGACAGCGATCC |
| expression vector | Am5-HT_2α_ | sense: TTTGCTAGCCACCATGGAGGGGGATCTGATCG |
|  |  | antisense: TTTGAATTCACAATTCTGATCGTGCTCG |
|  | Am5-HT_2β_ | sense: TTTAAGCTTCCACCATGATCGACTCGTCGACG |
|  |  | antisense: TTTACCGGTCCTGGACGGTCTCCAC |
| generation of antigen | Am5-HT_2α_ | sense: TTTGAATTCCGAGATCAATTCCGAAGG |
|  |  | antisense: TTTAAGCTTTTATTCACGGCCGATATTCTC |
